# The Real-World Early Neuroprotective Effects of Oral Citicoline Combination in Prodromal Dementia

**DOI:** 10.3390/nu18040595

**Published:** 2026-02-11

**Authors:** Aynur Özge, Ayhan Bingöl, Sevim Eyüboğlu, Ayşe İrem Can, Bahar Taşdelen, Ezgi Uluduz, Derya Uludüz

**Affiliations:** 1 Brain Health Clinic, Mersin 33010, Türkiye; aynurozge@gmail.com (A.Ö.); irem33.ic@gmail.com (A.İ.C.); 2 NOROM Neuroscience and Excellence Center, Ankara 06560, Türkiye; 3 Brain 360 Brain Health Clinic, Istanbul 34381, Türkiye; ayhan.bingol@yahoo.com.tr (A.B.); sevimeyupoglu@gmail.com (S.E.); 4 Department of Statistics, Mersin University Medical Faculty, Mersin 33343, Türkiye; bahartasdelen@gmail.com; 5 School of Medicine, Koc University, Istanbul 34450, Türkiye; ezgiuluduz@koc.edu.tr; 6 Neurology Department, Medical Faculty, Cerrahpasa University, Istanbul 34098, Türkiye

**Keywords:** citicoline, prodromal dementia, early Alzheimer’s disease, cognitive reserve, nutritional neuroprotection, executive function, memory, mood symptoms, observational study

## Abstract

**Background/Objectives:** Early intervention in the prodromal stages of dementia is a primary focus of contemporary research, as delaying clinical progression may have a substantial public health impact. Citicoline, an endogenous precursor of phosphatidylcholine and acetylcholine, has been proposed as a nutritional compound with potential relevance to multiple cognitive domains. However, real-world evidence regarding its specific contributions in prodromal dementia populations is limited. This study was conducted to examine cognitive, functional, and emotional outcomes associated with the use of an oral citicoline combined preparation in individuals with prodromal dementia and early Alzheimer’s type cognitive decline. **Methods:** This was a two-centre, retrospective, observational, real-world cohort study. A cohort of 100 patients receiving a combined oral citicoline preparation and 50 age-matched healthy controls were evaluated at baseline and followed for 6–9 months. Participants underwent comprehensive neuropsychological assessments that evaluated domains including executive function, attention, processing speed, working memory, visual-spatial and verbal memory, fluency, general cognition, and mood. Standardized instruments included Stroop indices, Trail Making Tests A/B, SDMT, SPART-based measures, SBST, fluency tasks, the Boston Naming Test, and MoCA. Statistical analyses included age-adjusted and education-level-stratified comparisons. **Results:** Use of the citicoline combined preparation was associated with improvements in several cognitive domains, including executive functions, processing speed, working memory, visual-spatial memory, and both semantic and episodic fluency (all p < 0.05). Functional memory scanning and global cognition also showed improvement over the observation period. Significant differences between groups were observed at baseline and follow-up for multiple cognitive indices (most p < 0.001). Mood outcomes were more favorable in the citicoline combined preparation group, with reductions in depressive and anxiety symptoms. Age-adjusted models identified age as an important covariate, and participants with lower educational levels demonstrated comparatively greater cognitive gains. **Conclusions:** In this real-world observational study, use of an oral citicoline combined preparation was associated with multidomain improvements in cognitive and mood-related outcomes in individuals with prodromal dementia/early Alzheimer-type decline. Given the observational design, these findings should be considered exploratory and require confirmation in prospective randomised controlled trials.

## 1. Introduction

The global increase in dementia prevalence has intensified interest in interventions that could alter the course of cognitive decline before irreversible neurodegeneration sets in. Growing evidence indicates that neuropathological changes associated with Alzheimer’s disease (AD) begin decades before clinical symptoms emerge, highlighting the importance of targeting the prodromal and even preclinical stages as a critical treatment window [1]. Mild cognitive impairment (MCI), often conceptualized as prodromal dementia, represents a transitional state where timely neuroprotective strategies may be associated with preservation of neuronal integrity and a slower trajectory toward overt dementia [2].

Citicoline (CDP-choline), an endogenous precursor of phosphatidylcholine and acetylcholine, is gaining attention for its multimodal neuroprotective and neurorestorative properties. Mechanistic studies show that citicoline is involved in processes related to neuronal membrane stability, increases phospholipid synthesis, improves mitochondrial efficiency, modulates dopaminergic and cholinergic neurotransmission, and reduces oxidative and inflammatory stress—all processes that are impaired in the earliest stages of neurodegeneration [3]. Preclinical and clinical studies reported associations between citicoline exposure and changes in phospholipid turnover, promoting neuroplasticity, and enhancing brain metabolism [4]. These mechanistic pathways have been recognized by regulatory agencies, including the European Food Safety Authority. The potential relevance of citicoline to cognitive performance, particularly for memory, is mechanistically plausible and biologically grounded [5]. Citicoline-based formulations combining phosphatidylserine, uridine monophosphate, and vitamins B1, B2, B6, and B12 are intended to provide a multimodal supportive strategy targeting complementary mechanisms implicated in early neurodegeneration. This combination may be relevant to neuronal membrane phospholipid biosynthesis and integrity, synaptic remodelling and plasticity, mitochondrial bioenergetic efficiency, and modulation of neurotransmitter systems relevant to memory and executive function. Therefore, in this retrospective study, the combined formulation was selected as a pragmatic real-world approach to improve synaptic resilience and cognitive performance in prodromal dementia. Clinical studies on age-related memory impairment, vascular cognitive impairment, and early Alzheimer’s-type decline suggest that citicoline use may be associated with better performance in attention, memory, and executive functions [6,7,8]. Observational data also support its potential relevance as an adjunct or standalone nutritional intervention in individuals with MCI or mixed neurodegenerative-vascular risk profiles [9,10]. Furthermore, citicoline has been proposed compound of interest for individuals at high genetic risk, particularly APOE ε4 carriers, in whom alterations in phospholipid metabolism contribute to early synaptic dysfunction [11].

Despite these promising findings, real-world evidence documenting the effects of citicoline, particularly in prodromal dementia and early Alzheimer ’s-type decline, is limited. Current studies differ in design, population characteristics, and outcome measures, leaving a gap in clinically applicable guidelines for routine practice [2,4]. Given the urgent need for early, safe, and accessible interventions, the systematic evaluation of citicoline combined preparation in real-world clinical settings is of great importance [3].

This retrospective observational study assessed real-world changes in cognitive and mood-related outcomes associated with the routine use of a combined oral citicoline-based formulation (Cogniven) in individuals with prodromal dementia or early Alzheimer-type cognitive decline. By integrating multi-domain neuropsychological data with real-world clinical follow-up, this study aims to clarify the potential role of citicoline as an early supportive nutritional approach. The present study was designed as a retrospective observational real-world analysis of clinical practice data.

## 2. Methods

Participants: This two-center retrospective observational real-world study includes data from 100 individuals with cognitive complaints receiving a combined oral citicoline-based formulation and 50 healthy controls who met the study criteria, drawn from 3690 patients followed up at specialized tertiary cognitive clinics. Diagnostic classifications within this group included Subjective Cognitive Decline (21%), Mild Cognitive Impairment (36%), early-stage Alzheimer-type cognitive decline (28%), moderate Alzheimer-type dementia (12%), and other dementia syndromes (3%). Within the MCI and early Alzheimer-type categories, approximately two-thirds presented with an amnestic single-domain profile, while one-third showed amnestic multi-domain involvement. All clinical assessments were performed by experienced neurologists (AO, DU). Diagnoses were classified according to the DSM-5 criteria [12] for neurocognitive disorders and the NIA–AA research framework [13] for Alzheimer’s disease.

Inclusion Criteria: Participants were included if they had a diagnosis within the prodromal/early Alzheimer-type spectrum according to DSM-5 and NIA–AA criteria; had complete baseline and follow up clinical records with a 6–9 month interval; received the combined citicoline with phosphatidylserine, uridine monophosphate, and vitamins B1, B2, B6, and B12; used the combined formulation as the primary supplement during the observation period.

Controls were included if they: had no neurological or psychiatric disease history, had no subjective cognitive complaints and no objective impairment during screening, and completed the same baseline assessment protocol.

**Figure 1:**
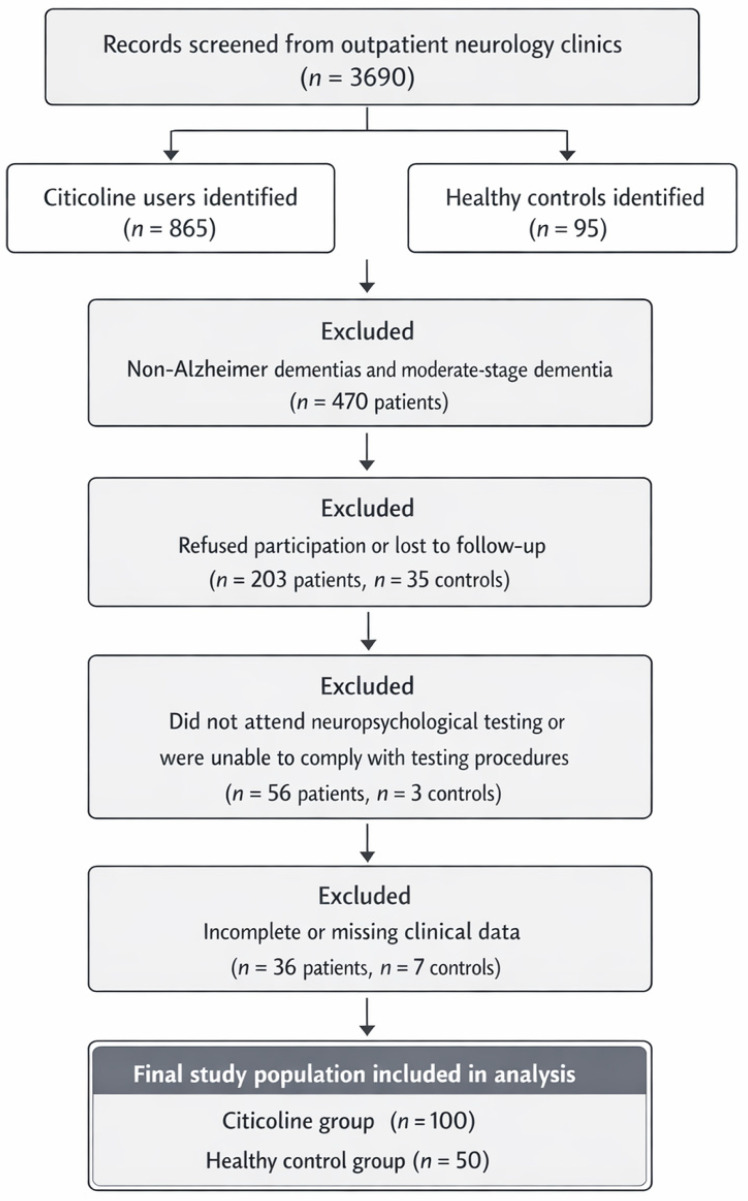
Flowchart of participant selection and study inclusion.

Exclusion Criteria: Participants were excluded if they: (1) were non-native speakers; (2) were illiterate; (3) had a history of major psychiatric disorders such as schizophrenia or obsessive–compulsive disorder; (4) had neurological conditions other than MCI or early-stage Alzheimer-type disorders; (5) were using medications known to interfere with cognitive performance; (6) had documented gastrointestinal malabsorption syndromes that could affect nutrient absorption or (7) did not adhere to the combined formulation for at least 6 months. All participants provided written informed consent for the use of their clinical data in research, in accordance with the standard operating procedures of both centres. The study was conducted in accordance with the Declaration of Helsinki, and approved by the Institutional Review Board (or Ethics Committee) of Toros University Ethics Committee (protocol code [5-49], date of approval [24 March 2022]). The citicoline-formulation cohort consisted of individuals who met established diagnostic criteria for prodromal or early-stage Alzheimer’s disease. Diagnostic classifications within this group included Subjective Cognitive Decline (21%), Mild Cognitive Impairment (36%), early-stage Alzheimer’s disease (28%), moderate Alzheimer’s-type dementia (12%), and other dementia syndromes (3%). The control group consisted of cognitively healthy adults who were matched as closely as possible for age and living environment and who were typically the patients’ spouses or first-degree relatives. All control participants were confirmed to have no history of neurological or psychiatric disorders and showed no signs of subjective or objective cognitive impairment during screening (see Figure 1, flow chart).

Neuroimaging and structural data acquisition: All patients in the citicoline-formulation cohort underwent structural brain MRI as part of the standardized tertiary-clinic diagnostic work-up. In addition, FDG-PET imaging was available for a subset of participants (n = 68), consistent with the distribution reported in Table 3.

Only neuroimaging examinations acquired and interpreted in an experienced tertiary imaging center by a qualified team were included. When scans retrieved from the national electronic health record system (https://enabiz.gov.tr/Account/Login?_st=1, accessed on 1 January 2023) did not meet these standardization and quality requirements, participants were requested to repeat the examination at the tertiary center. Hippocampal atrophy grading and Fazekas-based ratings of white matter hyperintensities were performed in accordance with the predefined standardized protocols used in our centers. 

The clinical use of the combined formulation evaluated in this manuscript reflects a real-world protocol for early-stage clinical support of individuals with prodromal dementia. Therefore, this retrospective analysis was not designed as a head-to-head comparative trial (e.g., citicoline alone vs. the combined formulation), but rather aimed to describe outcomes associated with the combined preparation as used in routine care.

Procedure: After diagnostic confirmation, participants with complete 6–9-month follow-up records were identified. Only individuals who had received oral citicoline combined with phosphatidylserine, uridine monophosphate, and vitamins B1, B2, B6, and B12 (Ocean Cogniven, Orzax Medicine Inc., İstanbul, Türkiye) as their exclusive cognitive-targeted intervention during this period were included in the intervention group. Participants received the combined formulation at a dosage of two capsules per day, providing a total daily citicoline dose of 500 mg, together with phosphatidylserine, uridine monophosphate, and vitamins B1, B2, B6, and B12. Only individuals who used the formulation continuously for at least 6 months were included. Baseline and follow-up cognitive evaluations were performed using a standardized 70-min neuropsychological battery administered in person by a licensed clinical psychologist. Given the study’s retrospective, real-world design and routine clinical documentation, assessors were aware of participants’ supplementation status. Narrative reports from each assessment were retained in the clinical archive. As a retrospective observational real-world analysis, the study was designed to describe outcomes observed in association with routine use of the combined formulation and does not permit causal inference regarding treatment effects

Demographic and clinical data—including comorbidities, concurrent medications, lifestyle factors, sensory deficits, and educational level—were systematically extracted from medical records using a standardized abstraction protocol. Educational attainment, a known modifier of cognitive performance, was categorized into three levels (elementary/middle school; high school; university/graduate school) and incorporated into all stratified and covariate analyses.

Participant flow, including inclusion and exclusion processes for both the citicoline combined preparation (citicoline group) and control cohorts, was documented using a PRISMA-adapted diagram (Figure 1) to ensure methodological transparency.

### 2.1. Neuropsychological Assessment

A harmonized neuropsychological test battery was administered across both centers to evaluate major cognitive domains commonly assessed in prodromal and early Alzheimer-type pathology, including episodic memory, verbal and visuospatial learning, working memory, processing speed, executive functions, language, and global cognition. Emotional state measures were included to account for potential mood-related confounding in cognitive performance. Documentation of the test battery is detailed in Supplement Table S1.

1. Brief repeatable battery of neuropsychological tests (BRB-N): This validated battery assessed visuospatial learning and memory (SPART and SPART-Delayed Recall), verbal learning and long-term retrieval (Selective Reminding Test and Delayed Recall), and processing speed/attention (Symbol Digit Modalities Test). Semantic and phonemic verbal fluency were evaluated via Word List Generation. These measures are considered sensitive to cognitive changes associated with hippocampal, parietal, and frontotemporal involvement in early neurodegenerative conditions. 

2. Executive function and attention measures: Executive control, selective attention, inhibition, and set-shifting were assessed using the Stroop Test and the Trail Making Test Parts A and B, which are widely used indicators of cognitive flexibility and information processing efficiency.

3. Global cognitive screening: Global cognition was evaluated using the Montreal Cognitive Assessment (MoCA), a widely used instrument covering executive, memory, visuospatial, language, abstraction, and orientation domains.

4. Additional cognitive measures: Language and semantic retrieval were assessed through the Boston Naming Test (short form). Visuospatial construction and planning were measured with the Clock Drawing Test. Mental sequencing and rapid attentional control were evaluated via the Mental Control subtest of the WMS-III.

5. Mood and anxiety measures: To address potential affective confounding, depressive and anxiety symptoms were assessed using the Beck Depression Inventory, Beck Anxiety Inventory, and the Geriatric Depression Scale, each with validated Turkish adaptations and established cutoffs. Mood assessments were included due to the well-documented association between affective symptoms and cognitive performance in prodromal dementia.

Within the MCI and early AD categories, the cohort was predominantly characterized by an amnestic cognitive profile. Specifically, most patients met criteria for a clinical phenotype consistent with amnestic MCI (aMCI), with episodic memory as the primary domain of impairment. In addition, a subset of individuals was classified as early-stage Alzheimer-type cognitive decline, primarily in the context of recent-onset, objectively documented memory impairment with preserved functional independence at presentation. Overall, the impairment pattern was largely consistent with an amnestic, mainly single-domain presentation, while a smaller proportion showed early involvement of additional cognitive domains, consistent with the expected clinical heterogeneity at the transition from aMCI to early AD.

### 2.2. Statistical Analysis

Continuous variables were assessed for normality using the Shapiro–Wilk test and summarized as mean ± SD. Baseline sociodemographic and lifestyle characteristics were compared using chi-square tests for categorical variables and Mann–Whitney U tests for continuous variables that were not normally distributed. Clinical comorbidities, medication profiles, and lifestyle risk factors were analyzed using chi-square or exact tests when appropriate.

Between-group differences in cognitive and mood change scores (Δ post–pre) were examined using Mann–Whitney U tests because the distributions were non-normal. To account for the modifying influence of cognitive reserve, all analyses were repeated across three education strata. Age-related variability was controlled using nonparametric ANCOVA (Quade’s test), and age-adjusted p-values (p_adj) were reported.

Statistical significance was defined as p < 0.05. Interpretation emphasized convergence across unadjusted, age-adjusted, and education-stratified models. All analyses were conducted using JASP (version 0.19.3; JASP Team, 2025).

## 3. Results

A total of 150 participants were included in the analysis: 100 individuals who received citicoline combined preparation and 50 cognitively healthy controls. Baseline demographic and lifestyle characteristics are summarized in Table 1. As expected for a prodromal cognitive cohort, the citicoline combined preparation group was significantly older than the control group (p < 0.001). There was a female majority in both groups. The time interval between baseline and follow-up neuropsychological assessments was calculated for all participants with complete longitudinal data. The mean follow-up duration was 7.3 ± 3.4 months (median: 6.4 months, range: 2.7–19.0 months). Only participants with both baseline and follow-up assessments were included in the analyses.

Primary outcomes included changes in global cognition (MoCA), executive functions (Trail Making Tests A/B and Stroop test), verbal and semantic fluency, episodic and working memory (WMS-III subtests), visuospatial memory (SPART and SPART-USB) and attention/processing speed (SDMT). Secondary outcomes assessed anxiety symptoms (Beck Anxiety Inventory) and functional memory performance (SBST and SBST-USB). This multidomain framework enabled a comprehensive evaluation of cognitive changes associated with the combined citicoline-based formulation across diagnostic and educational subgroups. Aside from age, baseline characteristics were largely comparable between groups; the only statistically significant lifestyle difference was a higher daily exposure to electronic screens in controls (p < 0.001). A trend toward a lower resting pulse rate was observed in the citicoline cohort (p = 0.053), while all other baseline variables were broadly similar between cohorts (Table 1). Therefore, subsequent analyses incorporated age adjustment and education stratification to minimize potential confounding.

All participants underwent baseline and follow-up neuropsychological assessments over a 6–9-month observation period. Primary outcomes assessed changes in global cognition and multiple cognitive domains (executive function, fluency, episodic/working memory, visuospatial memory, and attention/processing speed), while secondary outcomes included anxiety symptoms and functional memory performance.

Hypertension was significantly more prevalent in the citicoline cohort than in the control group (p = 0.007), and substantially more patients in the citicoline cohort were taking lipid-lowering therapy (p < 0.001). This reflects a greater cardiometabolic burden in this group. Hearing impairment was also markedly more prevalent in the citicoline group (p < 0.001). Conversely, regular physical activity was significantly more prevalent among the control group (p < 0.001), indicating a healthier lifestyle in those not taking citicoline. No other comorbidities, medication patterns or lifestyle risk factors differed significantly between the groups. These baseline differences were therefore accounted for in subsequent analyses to reduce potential confounding related to underlying cardiometabolic and sensory factors (see Table 2).

Neuroimaging findings are summarized in Table 3. Overall, structural imaging showed predominantly mild-to-moderate bilateral hippocampal atrophy accompanied by generally mild white-matter changes, with severe lesions observed only rarely. Among participants with available FDG-PET data, cerebral hypometabolism patterns were broadly consistent with Alzheimer-type neurodegeneration. APOE genotyping (where available) indicated a predominance of the E3/E3 genotype, with fewer carriers of E3/E4 or E4/E4 variants (Table 3).

Diagnostic profiling within the intervention cohort showed a heterogeneous distribution typical of real-world prodromal and early neurodegenerative presentations, including subjective cognitive decline, mild cognitive impairment, early Alzheimer-type cognitive decline, and less frequent atypical/vascular dementia syndromes. Low-frequency diagnostic categories were consolidated to ensure statistical stability in subgroup analyses. After adjustment for age, some effects diminished, underscoring that age is a strong covariate influencing cognitive performance. Still, consistent patterns favoured citicoline in memory- and fluency-related domains (see Table 4).

Education-stratified analyses suggested differential patterns of cognitive change across educational levels. In participants with lower educational attainment, significant positive changes were observed in visuospatial memory (SPART, p = 0.044; SPART-USB, p = 0.026) and functional memory scanning (SBST, p < 0.001). Global cognition improved in unadjusted analyses (MoCA, p < 0.001), although this association was attenuated after age adjustment. Episodic fluency also improved significantly in unadjusted analyses (p = 0.005) but did not remain significant following adjustment.

In the intermediate education group (high school), the citicoline cohort demonstrated significant improvements in processing speed (Trail Making Test A, p = 0.002; p_adj = 0.012), cognitive flexibility (Trail Making Test B, p = 0.020), working memory (WMS Serial 7s, p = 0.013), functional memory (SBST, p = 0.010), and episodic fluency (p = 0.007; p_adj = 0.043). However, several associations weakened after age adjustment, indicating a relevant influence of age on response patterns.

Participants with university or postgraduate education showed significant improvements in functional memory (SBST, p < 0.001; SBST-USB, p = 0.020) and global cognition (MoCA, p = 0.006) in unadjusted analyses. After age adjustment, significant gains were primarily observed in working memory, with WMS 3-digit (p_adj = 0.046) reaching significance. Overall, these findings suggest that educational level may modulate the cognitive domains most responsive to the combined citicoline-based formulation, with lower education showing stronger visuospatial/functional gains, and higher education showing improvements shifting toward working memory processes.

Education-stratified analyses suggested differential cognitive response patterns across educational levels (Table 4). Participants with lower educational attainment showed the greatest gains in visuospatial and functional memory scanning, with additional improvements in global cognition and verbal fluency. In the intermediate education group (high school), improvements were more evident in processing speed, cognitive flexibility, and working memory, alongside better functional memory performance. Participants with university or postgraduate education demonstrated a distinct response profile, with improvements shifting primarily toward functional memory and working memory–related processes.

Overall, fewer associations remained after age adjustment, suggesting that age was associated with variability in response patterns across educational strata (Table 4). The citicoline group showed measurable positive changes in executive functioning, episodic fluency, visuospatial memory, and functional memory scanning across all strata. Although age adjustment attenuated several results, consistent domain-specific patterns were observed: participants with lower education showed the largest positive changes in processing speed and cognitive flexibility, whereas those with higher education showed selective positive changes in working memory and learning efficiency. Together, these findings highlight an association between cognitive reserve and observed response pattern emphasising the importance of demographic stratification when interpreting outcomes of early-stage cognitive interventions.

Table 5 summarises baseline and follow-up performances on executive function, attention, processing speed and working memory measures. At baseline, the citicoline group demonstrated significantly lower performance than the control group on all Stroop indices, Trail Making Test Parts A and B, WMS Digit Span subtests, and the Symbol Digit Modalities Test (all p ≤ 0.013). This is consistent with a greater cognitive burden at the start of the study. At follow-up, although between-group differences remained statistically significant, the citicoline cohort showed consistent numerical improvements across multiple domains. These included reductions in Stroop completion time, faster performance on the Trail Making Test, and higher SDMT scores. These patterns indicate progressive enhancement in executive control, cognitive flexibility, and processing speed over the observation period. In contrast, the control group showed minimal change, maintaining relative cognitive stability. Overall, these results suggest that use of the combined citicoline-based formulation may be associated with positive changes in frontal executive and attentional processes, particularly in individuals with lower baseline cognitive performance.

Across all cognitive domains, the citicoline group demonstrated significantly poorer baseline performance than the control group (all p < 0.001), indicating a markedly poorer initial performance profile. Despite these persistent intergroup differences at follow-up, the citicoline group exhibited consistent within-group positive changes across key domains. Notable positive changes were observed in semantic and episodic fluency, visuospatial memory (SPART and SPART-USB), functional memory scanning (SBST), naming performance and global cognition (MoCA). Each of these showed higher median and mean follow-up scores than at baseline. Notably, there were also improvements in mood: depression scores were significantly lower than in the control group (p < 0.001), and anxiety scores showed a modest but significant reduction (p = 0.018). Taken together, these results suggest that use of the combined citicoline-based formulation was associated with quantifiable cognitive and emotional benefits to individuals with poorer initial performance, resulting in multidimensional improvements despite persistent performance disparities relative to the higher-functioning control group.

**Figure 2:**
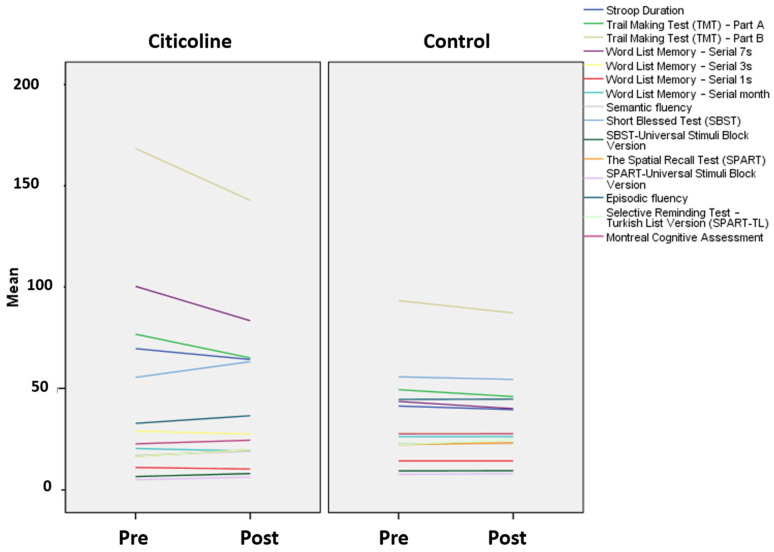
Pre–and post-changes in cognitive test performance in the citicoline and healthy control groups.

A domain-specific examination of cognitive outcomes revealed a broad pattern of improvement in the citicoline group over the 6–9-month follow-up period. Overall, positive changes were observed across multiple cognitive domains, most notably executive functioning and cognitive flexibility, attention/processing speed, and memory-related processes (working and episodic memory), alongside gains in verbal fluency and visuospatial learning and memory (see Table 4 and Table 5). Improvements were also evident in functional memory performance and global cognition, supporting a multidimensional pattern of change over the observation period. In contrast, the healthy control group exhibited fewer and more heterogeneous changes across domains, consistent with relative cognitive stability in the absence of the intervention (Table 4 and Table 5). When visualised longitudinally, the pre- and post-assessment trajectories clearly illustrate these patterns. Those receiving citicoline showed consistent improvements in executive functions, working memory, processing speed, verbal fluency, visuospatial memory, and global cognition. In contrast, the control group showed minimal change from baseline over time. Overall, these results highlight the potential of citicoline to deliver measurable, domain-specific cognitive benefits to individuals with early cognitive impairment, impacting multiple neurocognitive systems (Figure 2).

**Figure 3:**
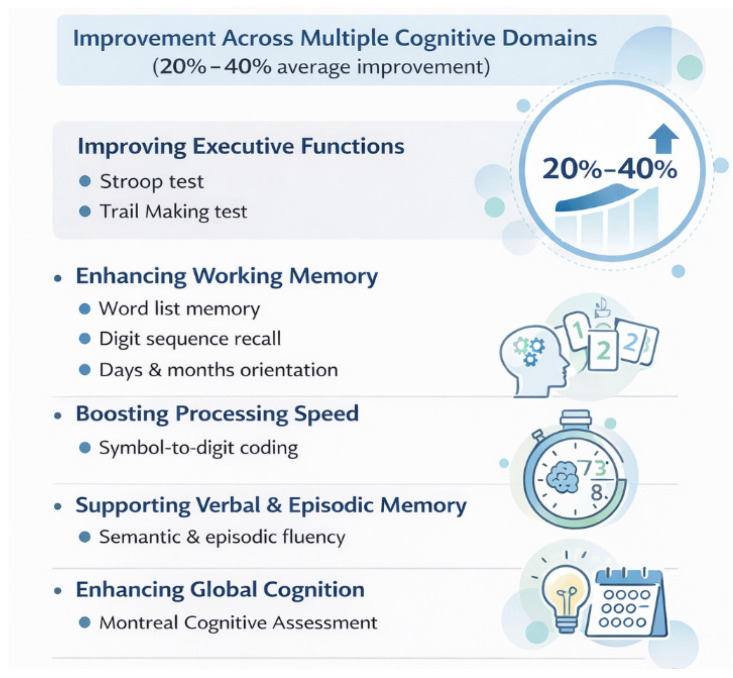
Summary of cognitive domain changes associated with citicoline treatment across supplementary analyses. Note: Significant unadjusted differences were observed; after age adjustment, only Trail Making A and Episodic Fluency remained significant.

In summary, citicoline was associated with measurable cognitive improvements across several domains, with effects varying by educational level see Figure 3 and Supplementary Figures.

## 4. Discussion

The citicoline-based formulation containing phosphatidylserine, uridine monophosphate, and vitamins B1, B2, B6, and B12 (Ocean Cogniven) has been developed based on biological rationales related to neuronal membrane integrity, synaptic plasticity, and neurochemical pathways essential for memory and executive processes. In this study, use of the formulation was associated with positive changes across several cognitive function parameters, suggesting a potential association with integrated neuroprotective and neurometabolic processes. This real-world, two-centre study suggests that a combined preparation of oral citicoline is associated with measurable, multidomain cognitive benefits in individuals at the prodromal stage of dementia. Observed response patterns appear to be associated with age and educational level. Through comprehensive neuropsychological assessments and stratified, age-adjusted analyses, the study identifies the cognitive systems most sensitive to citicoline. It further delineates patient subgroups in which clinically meaningful positive changes were most frequently observed. The diagnostic heterogeneity of the cohort reflects routine tertiary memory-clinic practice and supports the relevance of stratified analyses in real-world datasets. Such variability is expected in populations with prodromal and early cognitive decline and may contribute to domain-specific response patterns. Therefore, the observed cognitive changes should be interpreted as exploratory associations at the cohort level rather than as causal effects within a single homogeneous diagnostic entity. The observed improvements in neuropsychological test performance should be interpreted not only in terms of statistical significance but also in relation to their clinical magnitude and potential functional relevance. Although the effect sizes were generally in the small-to-moderate range, this level of change is considered clinically meaningful in populations with amnestic MCI and early-stage Alzheimer’s disease, where cognitive decline is typically progressive.

Citicoline provides choline and cytidine, which serve as substrates for phosphatidylcholine synthesis and acetylcholine production. Phosphatidylserine contributes directly to neuronal membrane integrity and has been associated with improvements in memory, attention, and executive function [14]. Uridine monophosphate has been associated with synaptogenesis and dendritic spine formation through effects on phospholipid biosynthesis and neuronal membrane turnover [15]. B vitamins (B1, B2, B6, B12) play established roles in mitochondrial energy metabolism, neurotransmitter synthesis, and homocysteine regulation, all of which influence cognitive performance [16].

Global cognition and executive–attention networks: Consistent between-group differences in global cognition (MoCA) and executive–attention measures support the relevance of citicoline in early cognitive decline. Positive changes in Trail Making Test A/B and Stroop performance are consistent with the documented effects of citicoline on frontostriatal and frontoparietal circuits, which depend on intact cholinergic and dopaminergic transmission, as well as phospholipid-dependent membrane stability [3,4]. These findings are consistent with earlier studies reporting enhanced processing speed and cognitive flexibility in MCI and early AD [2,8]. The partial attenuation of effects after age adjustment highlights the relevance impact of age-related neural constraints, emphasising the importance of early intervention before advanced network degeneration [1]. Even modest positive changes or stabilization in episodic memory and global cognitive scores may translate into preserved performance in memory-dependent activities of daily living, such as medication management, appointment tracking, and independent decision-making. In this context, the observed cognitive changes may reflect a slowing of expected decline or a short-term functional stabilization, which is of particular relevance for individuals at the transition from aMCI to early Alzheimer-type dementia.

Memory systems: Visuospatial, episodic, and working memory: Positive changes associated with citicoline use were particularly evident in visuospatial learning (SPART measures), functional memory scanning (SBST), and episodic fluency—domains closely linked to hippocampal–parietal circuits and synaptic membrane turnover. These positive changes are consistent with citicoline’s enhancement of phosphatidylcholine synthesis and mitochondrial efficiency [5,17]. Findings regarding working memory showed a selective pattern: higher-load tasks (e.g., WMS 7-digit) showed more apparent treatment effects, whereas overlearned sequences did not differ. This suggests that citicoline preferentially modulates attentional capacity rather than rote verbal recall [10].

Language and fluency: Significant positive changes in semantic and episodic fluency suggest that citicoline may support frontal–temporal retrieval processes, which are often impaired in the early stages of Alzheimer’s disease. These improvements are consistent with previous reports of enhanced fluency and lexical access associated with citicoline use in older adults [7,8]. The persistence of episodic fluency effects in lower-education subgroups suggests partial compensation for limited cognitive reserve.

Emotional and neuropsychiatric dimensions: Cognitive improvements were accompanied by reductions in depressive and anxiety symptoms, consistent with citicoline’s effects on monoaminergic pathways and frontal–limbic circuits [3]. Given the high prevalence of affective symptoms in prodromal dementia, these findings reinforce the bidirectional relationship between mood and cognitive performance.

Education, cognitive reserve, and treatment responsiveness: Stratified analyses revealed that individuals with lower educational attainment exhibited the strongest cognitive gains, particularly in processing speed, cognitive flexibility and episodic fluency, supporting the cognitive reserve model [2]. By contrast, highly educated participants demonstrated more selective improvements, particularly in working memory, after age adjustment. This suggests that positive changes associated with citicoline use in individuals with higher cognitive reserve may manifest as enhanced cognitive efficiency rather than broad performance changes.

From a clinical perspective, the magnitude of improvement observed in this study aligns with outcomes commonly reported for non-pharmacological or nutritional interventions in early cognitive impairment, supporting the potential role of such approaches as adjunctive strategies to maintain functional independence rather than achieve large-scale cognitive restoration.

### 4.1. Clinical Implications

The findings of this study have several important implications for the early management of cognitive decline in a clinical setting. Firstly, the multidomain positive changes observed in association with citicoline use, together with its favourable tolerability profile, support consideration of the combined formulation as a pragmatic nutritional adjunct in the supportive management of prodromal dementia, particularly in real-world clinical environments characterised by substantial heterogeneity in comorbidity burden, cognitive reserve, and lifestyle factors. The differential patterns of change observed across age and education groups highlight the importance of individualised supportive strategies rather than uniform intervention paradigms. It also suggests that citicoline could be particularly beneficial for individuals with lower baseline reserve or increased susceptibility to early cognitive decline.

Secondly, emerging evidence from large-scale, randomised, placebo-controlled trials of metabolic phenotypes (e.g., obesity, insulin resistance, and type 2 diabetes) provides a rigorous methodological framework for investigating the relationship between systemic metabolic optimisation and cognitive development. These trials emphasise the importance of metabolic profiling, encompassing inflammatory, mitochondrial, and lipid-regulatory pathways, as a key modifier of neurocognitive outcomes. Incorporating similar standardised metabolic modelling into future citicoline studies may help refine patient selection, strengthen mechanistic interpretation and enhance the translational relevance of findings.

Finally, the observed pattern of domain-specific responsiveness suggests that the potential neuroprotective effects of citicoline may be maximised if it is introduced during a narrow window of early network vulnerability. This is consistent with the broader clinical principle that interventions targeting phospholipid metabolism, synaptic turnover, and cholinergic/dopaminergic modulation may be more strongly associated with positive change when initiated before irreversible neurodegenerative changes occur. Taken together, these findings suggest that citicoline is a promising candidate for personalised, multimodal early intervention programmes for individuals at high risk of dementia.

### 4.2. Limitations

Several limitations should be considered when interpreting these findings. First, the absence of a randomized controlled trial and the observational design preclude causal inference; thus, the observed changes should be interpreted as associations in a real-world setting rather than as confirmatory evidence of efficacy. Although the real-world design enhances ecological validity, selection bias and confounding inherent to routine-care cohorts cannot be excluded. Additionally, because this study evaluated a combined formulation, the relative contributions of the individual components (citicoline, UMP, PS, and B vitamins) could not be disentangled. While age-adjusted analyses and education-stratified models were applied, residual confounding remains possible.

Secondly, baseline differences in demographic characteristics, including age and educational attainment, may have influenced cognitive performance. Although statistical adjustments reduced some of these effects, stratification revealed variability related to cognitive reserve; however, education is only a proxy for cognitive reserve and does not capture its multidimensional nature.

Third, the extensive neuropsychological battery increases the risk of type I error due to multiple comparisons. In line with the exploratory, hypothesis-generating nature of this study, no formal correction was applied. However, the consistent directionality of the results across related domains strengthens confidence in the overall pattern of findings.

Fourthly, lifestyle-related factors (dietary intake, sleep quality, physical activity) and concurrent supplement use were not systematically assessed, which may have contributed to inter-individual variability. Future studies integrating structured lifestyle characterization together with metabolic, inflammatory, and mitochondrial profiling may improve the interpretability of nutraceutical interventions [18].

Fifth, biomarker or neuroimaging confirmation of underlying pathology was not systematically available. Although clinical evaluation, neuropsychological assessment, and routine imaging are standard in real-world cohorts, the absence of amyloid/tau biomarkers and/or advanced neuroimaging confirmation may have introduced etiological heterogeneity, limiting pathology-specific conclusions.

In addition, standardized measures of functional independence (e.g., Instrumental Activities of Daily Living-IADL/Activities of Daily Living-ADL or Functional Independence Measure-FIM/Functional Assessment Measure-FAM) were not routinely collected in this retrospective real-world cohort. Therefore, we could not evaluate whether the observed cognitive changes translated into measurable improvements in everyday functioning. Future prospective studies should incorporate validated functional outcomes alongside neuropsychological measures.

Although the follow-up interval varied across participants, all assessments were conducted within a clinically meaningful early-intervention window, and the consistency of direction and magnitude of cognitive changes across domains suggests that the observed effects are unlikely to be driven solely by differences in follow-up duration. The 6–9-month follow-up period limits the ability to draw conclusions about long-term cognitive trajectories or potential disease-modifying effects. Insights from other chronic disease models, in which integrated phase III datasets inform dose thresholds, early-response criteria, and discontinuation strategies, highlight the need for protocolized citicoline trials to define optimal dosing, clinical use of the combined formulation duration, and early markers of responsiveness [19]. Longer-term, prospective, randomised, biomarker-informed studies are required to establish causality, delineate mechanisms and identify the optimal therapeutic window for the combined citicoline-based formulation

Because the study was designed to quantify continuous cognitive change in functionally preserved SCD, MCI, and early Alzheimer’s disease populations, formal cut-off–based reclassification of post-intervention ‘normalization’ versus residual impairment was not performed.

## 5. Conclusions

This study demonstrates that the investigated nutritional intervention is associated with statistically significant and clinically plausible improvements in cognitive performance among individuals with amnestic MCI and early-stage Alzheimer-type cognitive decline. Although the magnitude of change was modest, it is consistent with effect sizes reported for both existing nutritional strategies and currently available pharmacological treatments, particularly in early disease stages where large cognitive gains are rarely observed.

In contrast to pharmacological therapies, which primarily aim at symptomatic stabilization and may be limited by tolerability concerns, nutritional interventions offer a favourable safety profile and potential for sustained long-term use. From a clinical perspective, even small-to-moderate cognitive benefits may translate into meaningful functional implications, including preservation of memory-dependent daily activities and maintenance of functional independence during the prodromal and early phases of neurodegenerative disease.

Clinical and translational takeaway: In the current therapeutic landscape, nutritional interventions should be viewed as complementary strategies rather than alternatives to pharmacological treatment, contributing to a multimodal approach to slowing cognitive decline and supporting everyday functioning in early cognitive impairment. These findings support the integration of evidence-based nutritional approaches into personalized care pathways for individuals at risk of progression from MCI to Alzheimer’s disease.

## Figures and Tables

**Table 1:** Baseline sociodemographic and lifestyle characteristics of the study groups.

Variable	Citicoline Group (n = 100)	Control Group (n = 50)	p-Value
**Age, years**	67.13 ± 7.93	50.86 ± 4.30	<0.001
**Female sex, n (%)**	63 (63.0)	39 (78.0)	0.063
Education *			0.053
**─Primary school**	27 (29.3)	12 (27.3)	
**─Middle school**	8 (8.7)	1 (2.3)	
**─High school**	19 (20.7)	11 (25.0)	
**─University**	38 (41.3)	17 (38.6)	
**─Postgraduate**	0 (0.0)	3 (6.8)	
**Caffeine intake, mg/day**	291.21 ± 221.28	282.91 ± 204.58	0.878
**Resting pulse, beats/min**	73.38 ± 12.83	80.39 ± 14.62	0.053
**Oxygen saturation, %**	96.71 ± 2.58	97.35 ± 2.60	0.398
**Daily water intake, L/day**	1.47 ± 0.58	1.58 ± 0.71	0.618
**Electronic screen exposure, h/day**	4.03 ± 2.96	5.32 ± 3.64	<0.001

* Education data available for 92 individuals in the citicoline group and 44 in the control group. Data are presented as mean ± standard deviation or n (%). p-values are derived from independent-samples *t* tests or χ^2^ tests, as appropriate. Only participants with complete baseline and follow-up neuropsychological assessments were included in the analyses; therefore, there were no missing data for cognitive outcome measures, and no imputation procedures were required.

**Table 2:** Clinical comorbidities, medication use, and lifestyle risk factors in the citicoline and control groups.

Variable	Citicoline Group n (%)	Control Group n (%)	p-Value
**Multivitamin**	22 (22.0)	8 (16.0)	0.517
**Huperzine**	0 (0.0)	1 (2.0)	0.333
**Omega-3 fatty acids**	15 (15.0)	5 (10.0)	0.456
**Hypertension**	40 (40.0)	9 (18.0)	0.007
**Diabetes mellitus**	23 (23.0)	6 (12.0)	0.108
**Dyslipidemia**	32 (32.0)	14 (28.0)	0.616
**MI history**	2 (2.0)	0 (0.0)	NC
**Cancer history**	3 (3.0)	1 (2.0)	0.714
**COVID-19 history**	7 (7.0)	1 (2.0)	0.270
**PPI use**	9 (9.0)	4 (8.0)	0.837
**Lipid-lowering therapy**	27 (27.0)	1 (2.0)	<0.001
**Cholecystectomy**	3 (3.0)	3 (6.0)	0.401
**Depression history**	27 (27.0)	13 (26.0)	0.896
**Anxiety disorders**	20 (20.0)	11 (22.0)	0.776
**Stroke/TIA**	5 (5.0)	0 (0.0)	0.170
**Regular physical activity**	8 (8.0)	19 (38.0)	<0.001
**Smoking**	11 (11.0)	4 (8.0)	0.564
**Alcohol use**	8 (8.0)	3 (6.0)	0.658
**Hearing impairment**	24 (24.0)	0 (0.0)	<0.001
**Sleep quality (poor)**	6 (6.0)	7 (14.0)	0.101

Data are presented as n (%). p-values are derived from χ^2^ tests or Fisher’s exact tests as reported in the original analysis output. NC = not calculated because of zero or empty cells in contingency tables.

**Table 3:** Neuroimaging, Genetic, and Metabolic Characteristics of the Study Population (n = 100).

Variable	Category/Grade	n (%)
**Right hippocampus**	0	16 (16.0)
	1	57 (57.0)
	2	25 (25.0)
	3	2 (2.0)
**Left hippocampus**	0	20 (20.0)
	1	36 (36.0)
	2	40 (40.0)
	3	4 (4.0)
**Volumetric hippocampal atrophy**	0	12 (12.0)
	1	42 (42.0)
	2	39 (39.0)
	3	7 (7.0)
**Periventricular white matter changes**	0	50 (50.5)
	1	43 (43.4)
	2	5 (5.1)
	Severe	1 (1.0)
**Subcortical white matter changes**	0	34 (34.0)
	1	49 (49.0)
	2	17 (17.0)
**APOE genotype**	E3/E3	26 (57.8)
	E3/E4	15 (33.3)
	E4/E4	4 (8.9)
**Hypometabolism (FDG-PET)**	Mild	58 (85.3)
	Moderate	10 (14.7)
**Hypometabolism location**	Frontotemporal	11 (78.6)
	Subcortical	3 (21.4)

**Table 4:** Group Differences in Cognitive Change Scores (Δ Score = Post–Pre).

Cognitive Measure	Citicoline Mean ± SD	Control Mean ± SD	p-Value	p_adj (Age-Adjusted)
**MoCA**	1.35 ± 3.49	0.02 ± 1.67	<0.001	0.144
**Stroop Time (s)**	−7.62 ± 31.91	−1.20 ± 12.55	0.029	0.548
**Stroop Errors**	−0.12 ± 1.60	−0.14 ± 0.67	0.907	0.376
**Stroop Spontaneous**	−0.40 ± 2.80	−0.32 ± 1.06	0.434	0.585
**Abstract Reasoning**	0.05 ± 1.47	0.26 ± 0.92	0.353	0.156
**Trail Making Test–A**	−9.90 ± 26.13	−2.86 ± 13.75	0.075	0.665
**Trail Making Test–B**	−14.23 ± 48.13	−6.68 ± 20.23	0.464	0.457
**WMS Serial 7s**	−15.39 ± 58.69	−3.18 ± 14.95	0.055	0.377
**WMS Serial 3s**	−3.78 ± 23.04	−0.06 ± 47.53	0.142	0.882
**WMS Immediate (1-digit)**	−0.65 ± 5.02	0.00 ± 9.75	0.644	0.845
**WMS Week Recall**	−0.21 ± 5.32	0.90 ± 10.51	0.543	0.778
**WMS Month Recall**	−1.06 ± 15.34	0.00 ± 35.34	0.657	0.924
**SDMT**	3.93 ± 9.80	2.74 ± 7.95	0.655	0.350
**Semantic Fluency**	1.57 ± 4.52	0.10 ± 3.72	0.075	0.876
**SBST**	5.55 ± 9.24	−0.22 ± 5.33	<0.001	0.360
**SBST-USB**	1.13 ± 2.44	0.26 ± 1.96	0.049	0.868
**SPART**	2.62 ± 4.31	0.60 ± 3.46	0.044	0.742
**SPART-USB**	0.97 ± 1.96	0.26 ± 1.41	0.026	0.521
**Episodic Fluency**	2.87 ± 9.03	0.22 ± 5.01	0.005	0.113
**Boston Naming Test**	0.32 ± 4.16	0.00 ± 0.70	0.237	0.569
**SPART-TL**	2.62 ± 4.35	2.54 ± 3.42	0.412	0.340
**Beck Anxiety Inventory**	−0.41 ± 5.06	−0.26 ± 4.79	0.507	0.302

Note: Δ scores represent post–pre differences. p_adj values were computed using nonparametric ANCOVA controlling for age.

**Table 5:** Executive functions, attention, processing speed, and working memory.

Variable	Citicoline (n = 100) Median (Min–Max)	Citicoline Mean ± SD	Control (n = 50) Median (Min–Max)	Control Mean ± SD	p-Value
**Stroop Duration—Baseline**	65 (10–211)	74.18 ± 34.11	39 (14–62)	38.68 ± 8.27	<0.001
**Stroop Duration—Follow-up**	60 (12–180)	69.33 ± 31.83	38.5 (14–59)	37.48 ± 7.75	<0.001
**Stroop Errors—Baseline**	0 (0–8)	1.06 ± 1.54	0 (0–2)	0.38 ± 0.57	0.013
**Stroop Errors—Follow-up**	0 (0–8)	0.97 ± 1.55	0 (0–1)	0.24 ± 0.43	0.010
**Stroop Spontaneous—Baseline**	2 (0–14)	2.80 ± 2.90	1 (0–3)	1.06 ± 0.82	<0.001
**Stroop Spontaneous—Follow-up**	2 (0–14)	2.47 ± 2.85	1 (0–3)	0.74 ± 0.78	<0.001
**Trail Making Test A—Baseline (s)**	61.5 (30–265)	77.02 ± 40.84	48 (25–67)	48.78 ± 9.72	<0.001
**Trail Making Test A—Follow-up (s)**	56 (25–206)	67.12 ± 32.99	45 (24–64)	45.92 ± 8.92	<0.001
**Trail Making Test B—Baseline (s)**	150 (62–380)	176.51 ± 75.79	93 (59–135)	91.16 ± 15.36	<0.001
**Trail Making Test B—Follow-up (s)**	134.5 (60–320)	154.31 ± 61.84	87 (35–112)	84.48 ± 14.38	<0.001
**WMS Digit Span 7—Baseline**	86 (15–249)	105.9 ± 57.76	38.5 (17–68)	40.18 ± 11.74	<0.001
**WMS Digit Span 7—Follow-up**	75 (16–220)	88.20 ± 45.76	35 (16–60)	37.00 ± 10.56	<0.001
**SDMT—Baseline**	36 (6–73)	35.19 ± 11.89	58.5 (51–74)	58.64 ± 4.98	<0.001
**SDMT—Follow-up**	40 (8–79)	39.52 ± 11.27	61.5 (44–72)	61.38 ± 6.08	<0.001

Values are presented as median (min–max) and mean ± SD. Between-group comparisons were performed using the Mann–Whitney U test.

## Data Availability

The data are not publicly available due to privacy and ethical restrictions but are available from the corresponding author upon reasonable request.

## Supplementary Materials

The following supporting information can be downloaded at: https://www.mdpi.com/article/10.3390/nu18040595/s1, Figure S1. (**a**). Changes in Stroop Test Duration According to Educational Level in Citicoline and Control Groups. (**b**). Changes in Stroop Test Error Scores According to Educational Level in Citicoline and Control Groups. (**c**). Changes in Stroop Spontaneous Response Scores According to Educational Level in Citicoline and Control Groups. (**d**). Changes in Stroop Evaluation Scores According to Educational Level in Citicoline and Control Groups. Figure S2. (**a**). Changes in Trail Making Test Part A Completion Time According to Educational Level in Citicoline and Control Groups. (**b**). Changes in Trail Making Test Part B Completion Time According to Educational Level in Citicoline and Control Groups. Figure S3. (**a**). Changes in Word List Memory (Serial 7s) Scores According to Educational Level in Citicoline and Control Groups. Figure (**b**). Changes in Word List Memory (Serial 3s) Scores According to Educational Level in Citicoline and Control Groups. (**c**). Changes in Word List Memory (Serial 1s) Scores According to Educational Level in Citicoline and Control Groups. (**d**). Changes in Word List Memory (Days of a Week) Scores According to Educational Level in Citicoline and Control Groups. (**e**). Changes in Word List Memory (Months of the Year) Scores According to Educational Level in Citicoline and Control Groups. Figure S4. Changes in Symbol Digit Modalities Test (SDMT) Scores According to Educational Level in Citicoline and Control Groups. Figure S5. Changes in Semantic Fluency Scores According to Educational Level in Citicoline and Control Groups. Figure S6. Changes in Short Blessed Test (SBST) Scores According to Educational Level in Citicoline and Control Groups. Figure S7. Changes in Short Blessed Test—Universal Stimuli Block Version (SBST-USB) Scores According to Educational Level in Citicoline and Control Groups. Figure S8. Changes in SPART—Universal Stimuli Block Version Scores According to Educational Level in Citicoline and Control Groups. Figure S9. Changes in Episodic Fluency Scores According to Educational Level in Citicoline and Control Groups. Figure S10. Changes in Boston Naming Test (BNT) Scores According to Educational Level in Citicoline and Control Groups. Figure S11. Changes in SPART Total Learning (SPART-TL) Scores According to Educational Level in Citicoline and Control Groups. Table S1. Documentation of the test battery. Table S2. Memory, language, global cognition, and emotional measures.

## References

[1] Bonvicini M., Travaglini S., Lelli D., Antonelli Incalzi R., Pedone C. (2023). Is citicoline effective in preventing and slowing down dementia? A systematic review and a meta-analysis. Nutrients.

[2] Bermejo P.E., Dorado R., Zea-Sevilla M.A. (2023). Role of citicoline in patients with mild cognitive impairment. Neurosci. Insights.

[3] Gareri P., Cotroneo A.M., Montella R., Gaglianone M., Putignano S. (2024). Citicoline: A cholinergic precursor with a pivotal role in dementia and Alzheimer’s disease. J. Alzheimer’s Dis..

[4] Secades J.J., Gareri P. (2022). Citicoline: Pharmacological and clinical review, 2022 update. Rev. Neurol..

[5] Turck D., Bohn T., Castenmiller J., De Henauw S., Hirsch-Ernst K.I., Knutsen H.K., Maciuk A., Mangelsdorf I., McArdle H.J., Naska A. (2024). “Citicoline” and support of the memory function: EFSA evaluation. EFSA J..

[6] Álvarez-Sabín J., Román G.C. (2011). Citicoline in vascular cognitive impairment and vascular dementia. Stroke.

[7] Nakazaki E., Mah E., Sanoshy K., Citrolo D., Watanabe F. (2021). Citicoline and memory function in healthy older adults. J. Nutr..

[8] Swiatkiewicz M., Grieb P. (2023). Citicoline for supporting memory in aging humans. Aging Dis..

[9] Castagna A., Fabbo A., Manzo C., Lacava R., Ruberto C., Ruotolo G. (2021). A retrospective study on the benefits of combined citicoline, memantine, and acetylcholinesterase inhibitor treatments in older patients affected with Alzheimer’s disease. J. Alzheimer’s Dis..

[10] Gareri P., Cotroneo A.M., Orsitto G., Putignano S. (2020). The CITIMEM study: A pilot study. Optimizing pharmacological treatment in dementia. Arch. Gerontol. Geriatr..

[11] Selezneva N.D., Gavrilova S.I., Roshchina I.F., Ponomareva E.V. (2021). Citicoline in cognitive impairment in first-degree relatives of AD patients. Zh. Nevrol. Psikhiatr. Im. S.S. Korsakova.

[12] American Psychiatric Association (2013). Diagnostic and Statistical Manual of Mental Disorders (DSM-5).

[13] Jack C.R., Bennett D.A., Blennow K., Carrillo M.C., Dunn B., Haeberlein S.B., Holtzman D.M., Jagust W., Jessen F., Karlawish J. (2018). NIA-AA research framework: Toward a biological definition of Alzheimer’s disease. Alzheimer’s Dement..

[14] Baumel B.S., Doraiswamy P.M., Sabbagh M., Wurtman R. (2021). Potential Neuroregenerative and Neuroprotective Effects of Uridine/Choline-Enriched Multinutrient Dietary Intervention for Mild Cognitive Impairment: A Narrative Review. Neurol. Ther..

[15] Wurtman R.J., Ulus I.H., Cansev M., Watkins C.J., Wang L., Marzloff G. (2006). Synaptic proteins and phospholipids are increased in gerbil brain by administering uridine plus docosahexaenoic acid orally. Brain Res..

[16] Glade M.J., Smith K. (2015). Phosphatidylserine and the human brain. Nutrition.

[17] Secades J.J. (2019). Citicoline in the treatment of cognitive impairment. J. Neurol. Exp. Neurosci..

[18] Nederveen J.P., Mastrolonardo A.J., Xhuti D., Di Carlo A., Manta K., Fuda M.R., Tarnopolsky M.A. (2023). Novel multi-ingredient supplement facilitates weight loss and improves body composition in overweight and obese individuals: A randomized, double-blind, placebo-controlled clinical trial. Nutrients.

[19] Jemec G.B.E., Okun M.M., Forman S.B., Gulliver W.P.F., Prens E.P., Mrowietz U., Armstrong A.W., Geng Z., Gu Y., Williams D.A. (2019). Adalimumab medium-term dosing strategy in moderate-to-severe hidradenitis suppurativa: Integrated results from the phase III randomized placebo-controlled PIONEER trials. Br. J. Dermatol..

