# Supplementary material for: The Real-World Early Neuroprotective Effects of Oral Citicoline Combination in Prodromal Dementia
Source: Nutrients. 2026 Feb 11;18(4):595. doi: 10.3390/nu18040595 (PMC12942905; doi:10.3390/nu18040595)
Supplement: Supplementary file 1 [file nutrients-18-00595-s001.zip › nutrients-4094369-supplementary.pdf]

Supplementary figures;

Figure S1. Detail comparisons of Stroop tests between citicoline and control groups.

Figure S1a. Changes in Stroop Test Duration According to Educational Level in Ci ticoline and Control Groups.

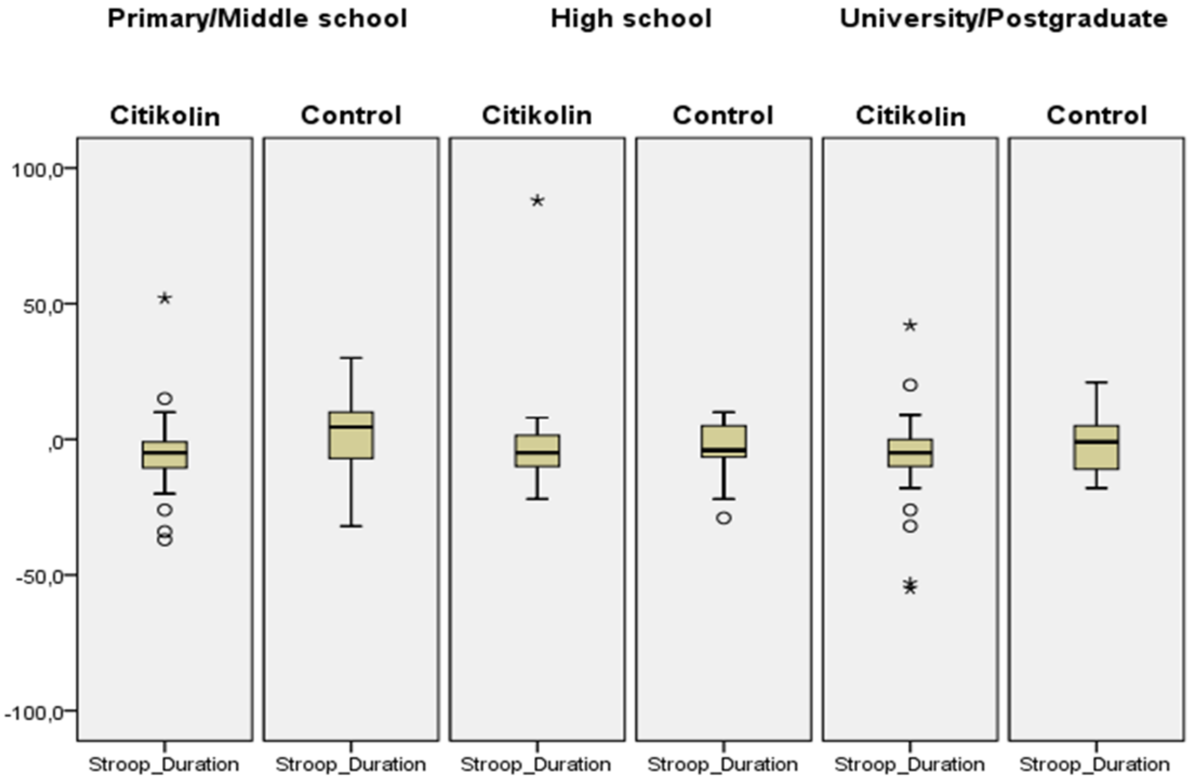

Figure S1b. Changes in Stroop Test Error Scores According to Educational Level in Ci ticoline and Control Groups.

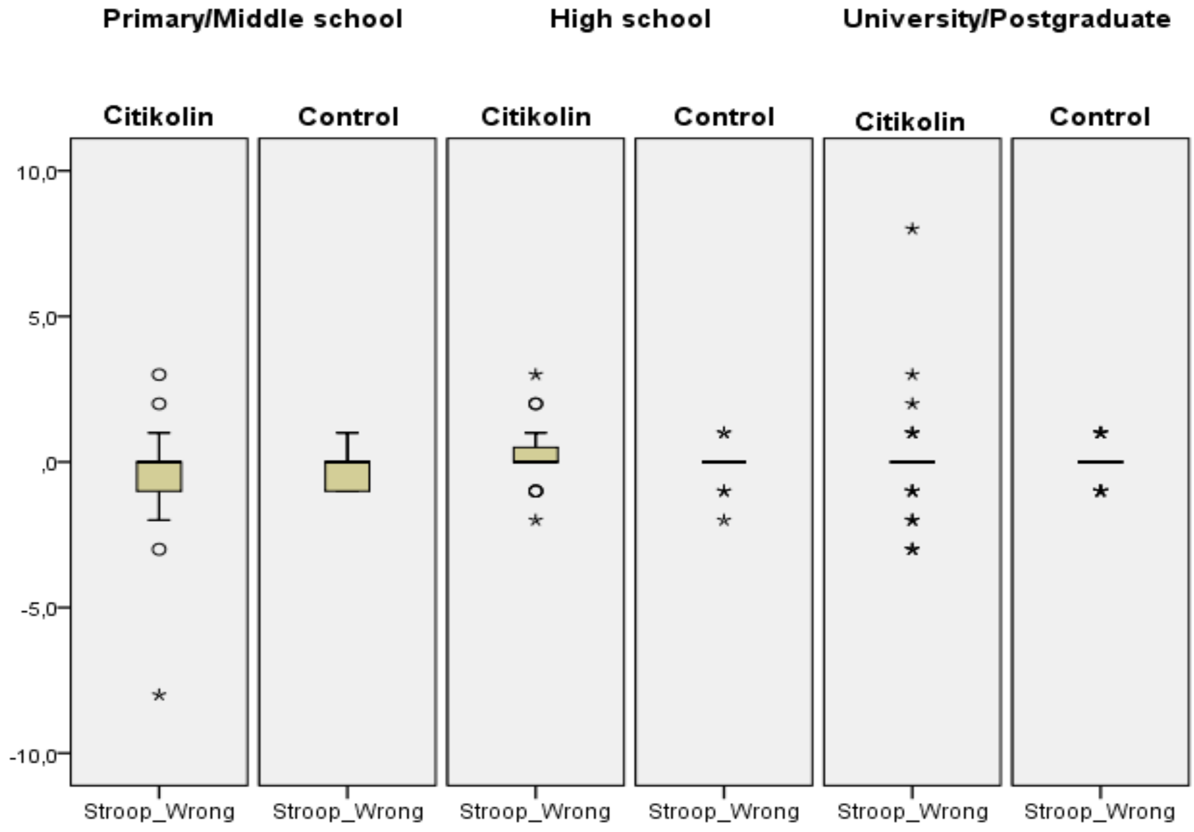

Figure S1c. Changes in Stroop Spontaneous Response Scores According to Educa tional Level in Citicoline and Control Groups.

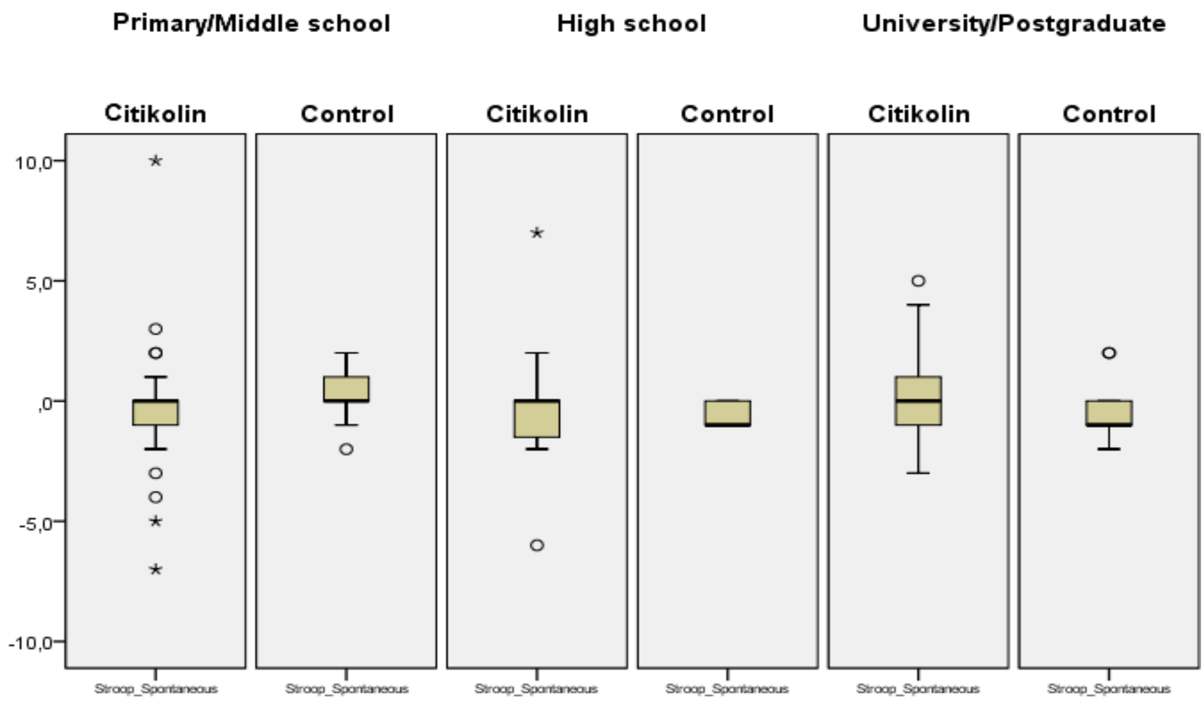

Figure S1d. Changes in Stroop Evaluation Scores According to Educational Level in Ci ticoline and Control Groups.

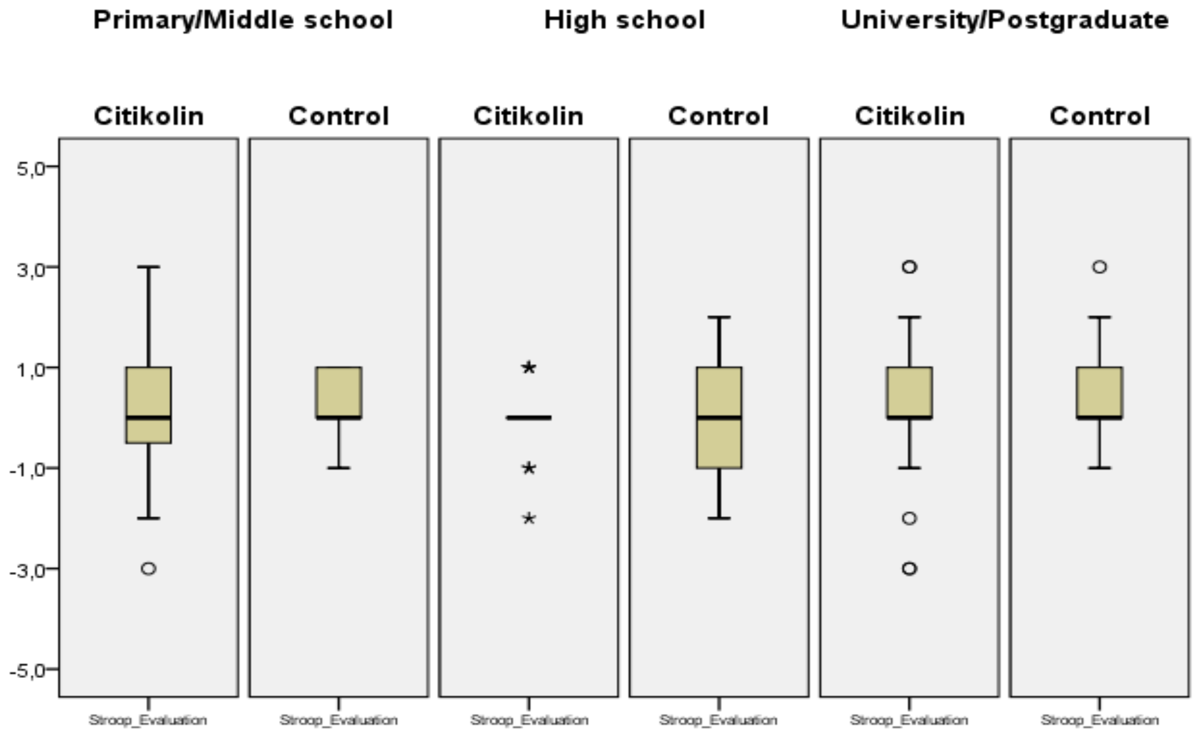

Figure S2a. Changes in Trail Making Test Part A Completion Time According to Educa tional Level in Citicoline and Control Groups.

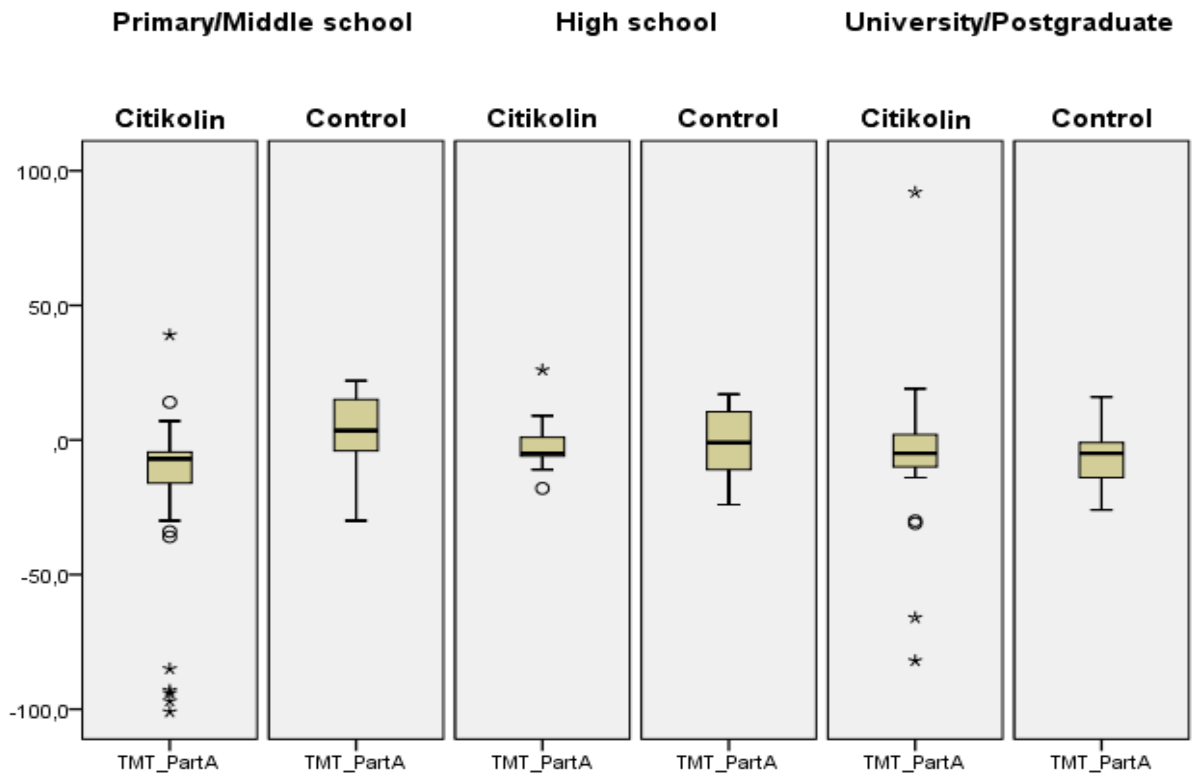

Figure S2b. Changes in Trail Making Test Part B Completion Time According to Educa tional Level in Citicoline and Control Groups.

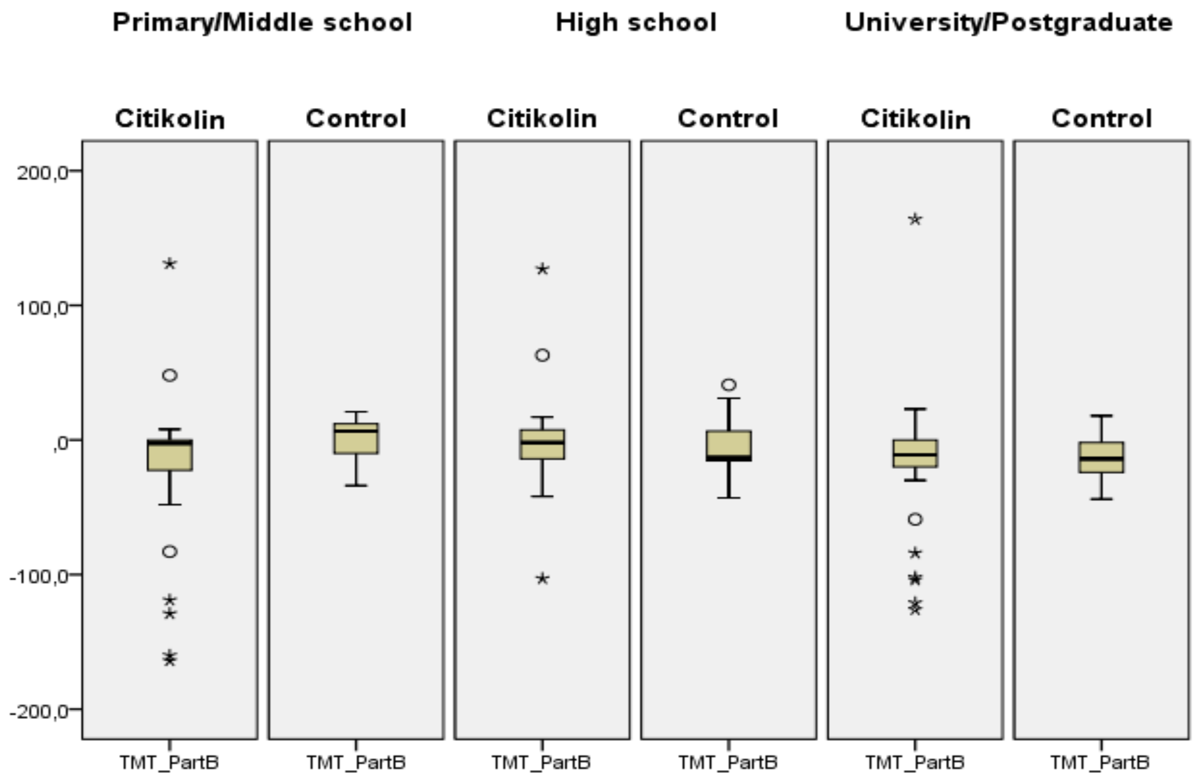

Figure S3a. Changes in Word List Memory (Serial 7s) Scores According to Educatio nal Level in Citicoline and Control Groups.

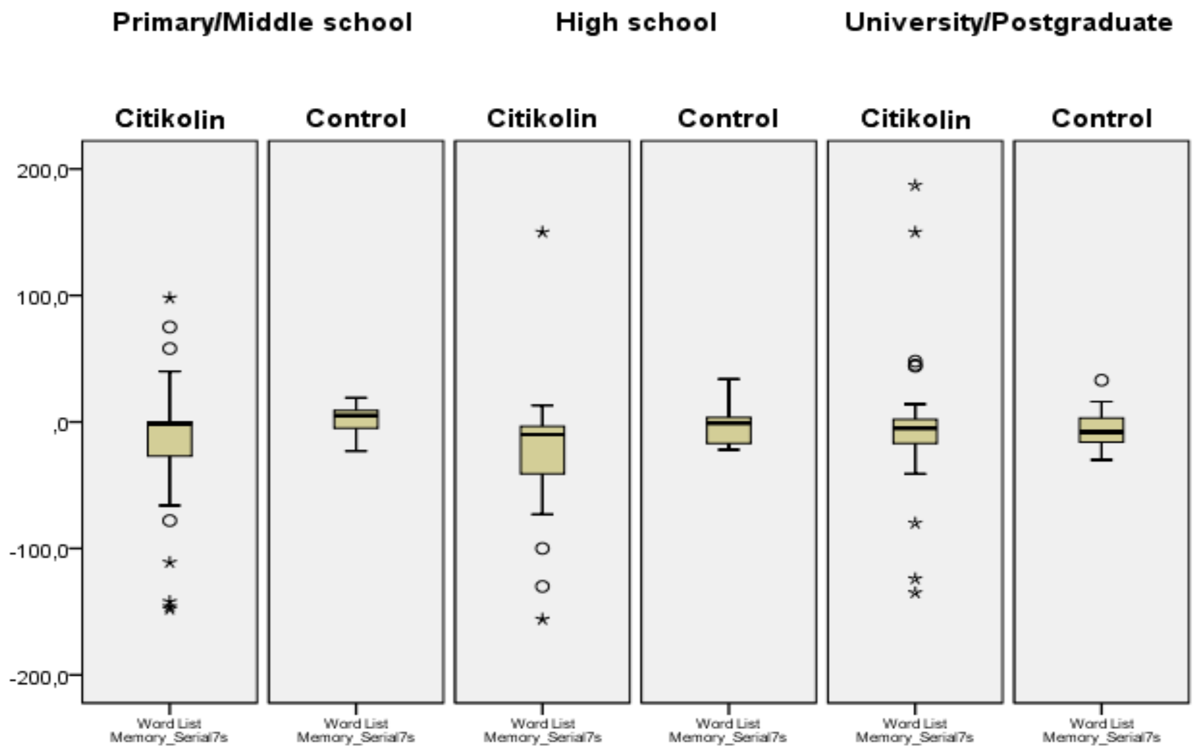

Figure S3b. Changes in Word List Memory (Serial 3s) Scores According to Educa tional Level in Citicoline and Control Groups.

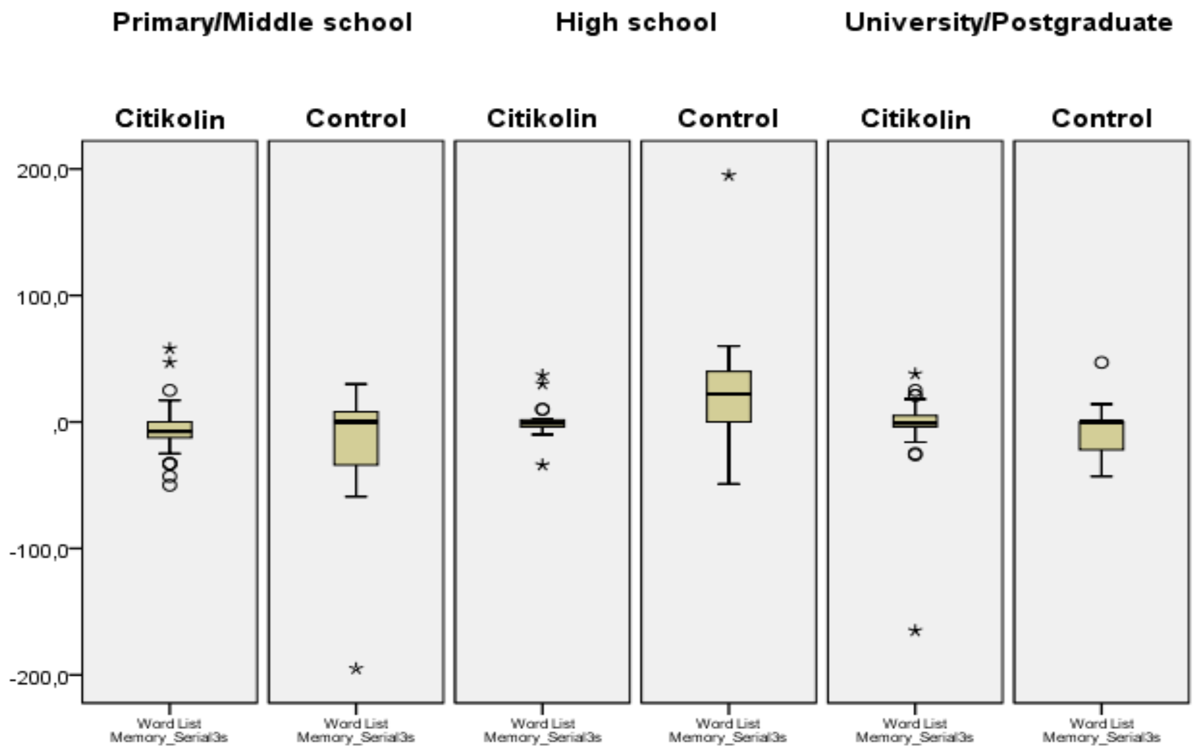

Figure S3c. Changes in Word List Memory (Serial 1s) Scores According to Educa tional Level in Citicoline and Control Groups.

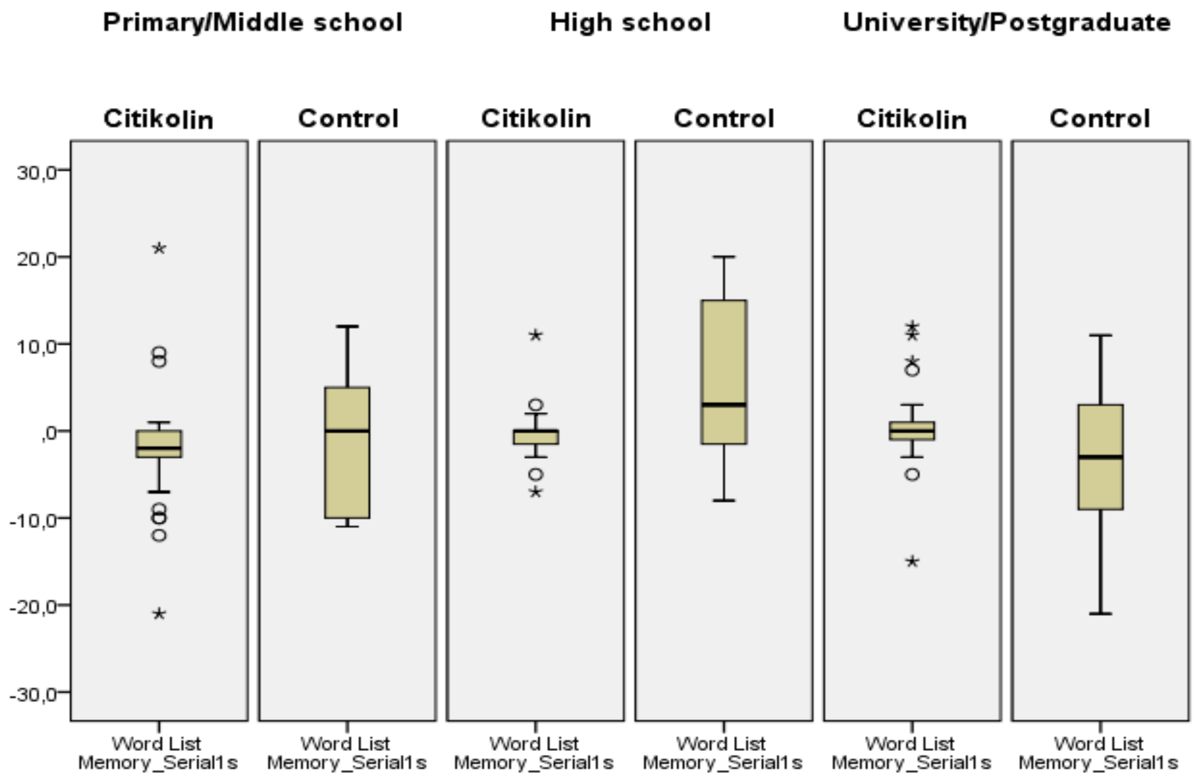

Figure S3d. Changes in Word List Memory (Days of a Week) Scores According to Educa tional Level in Citicoline and Control Groups.

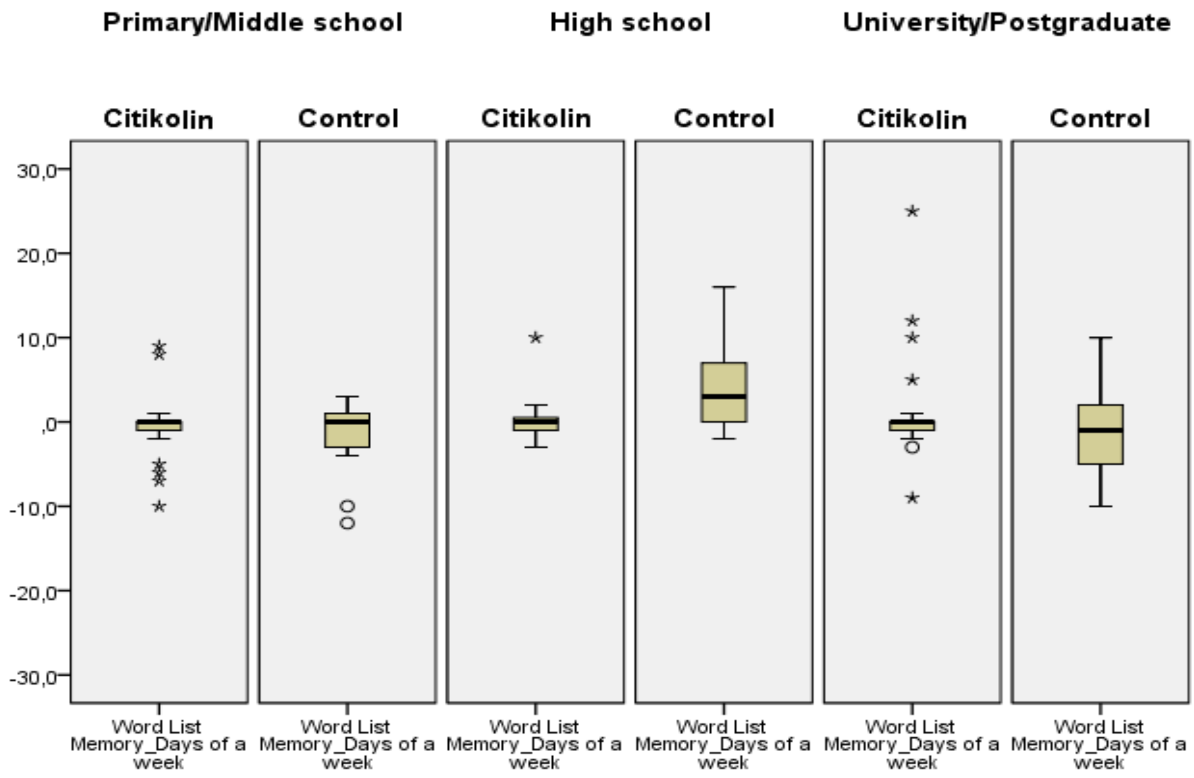

Figure S3e. Changes in Word List Memory (Months of the Year) Scores According to Educational Level in Citicoline and Control Groups.

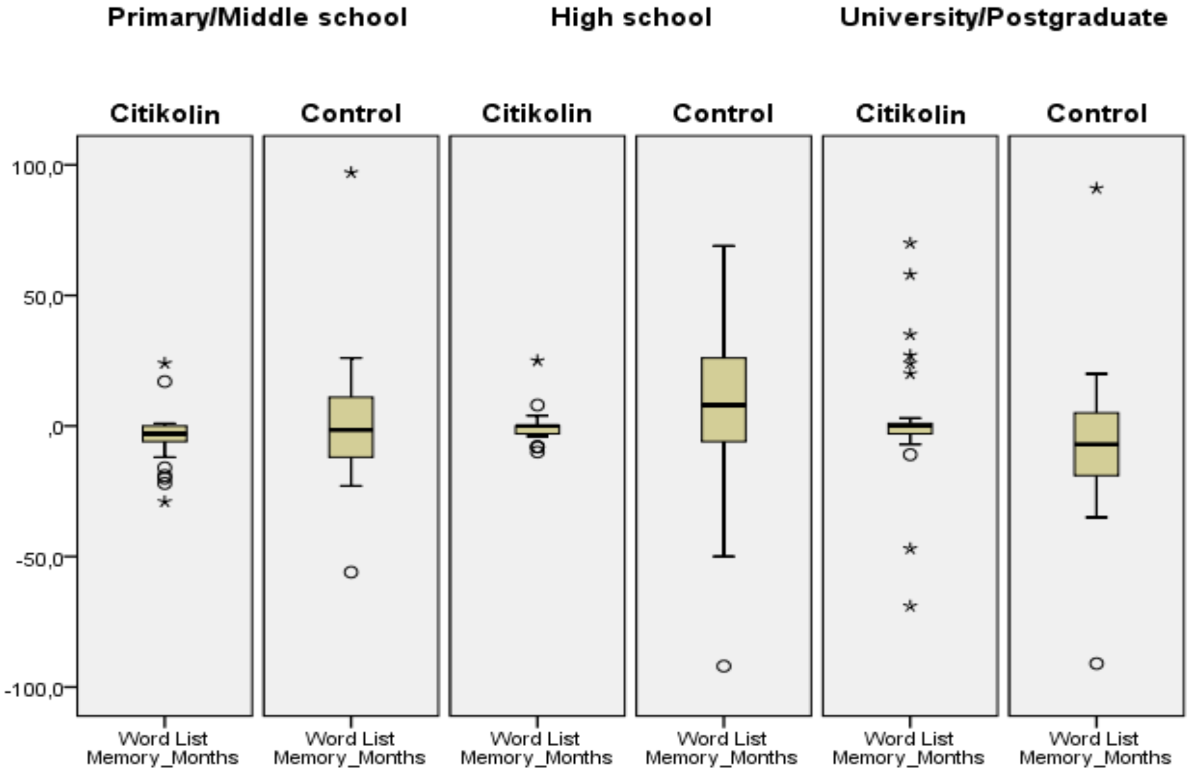

Figure S4. Changes in Symbol Digit Modalities Test (SDMT) Scores According to Educa tional Level in Citicoline and Control Groups.

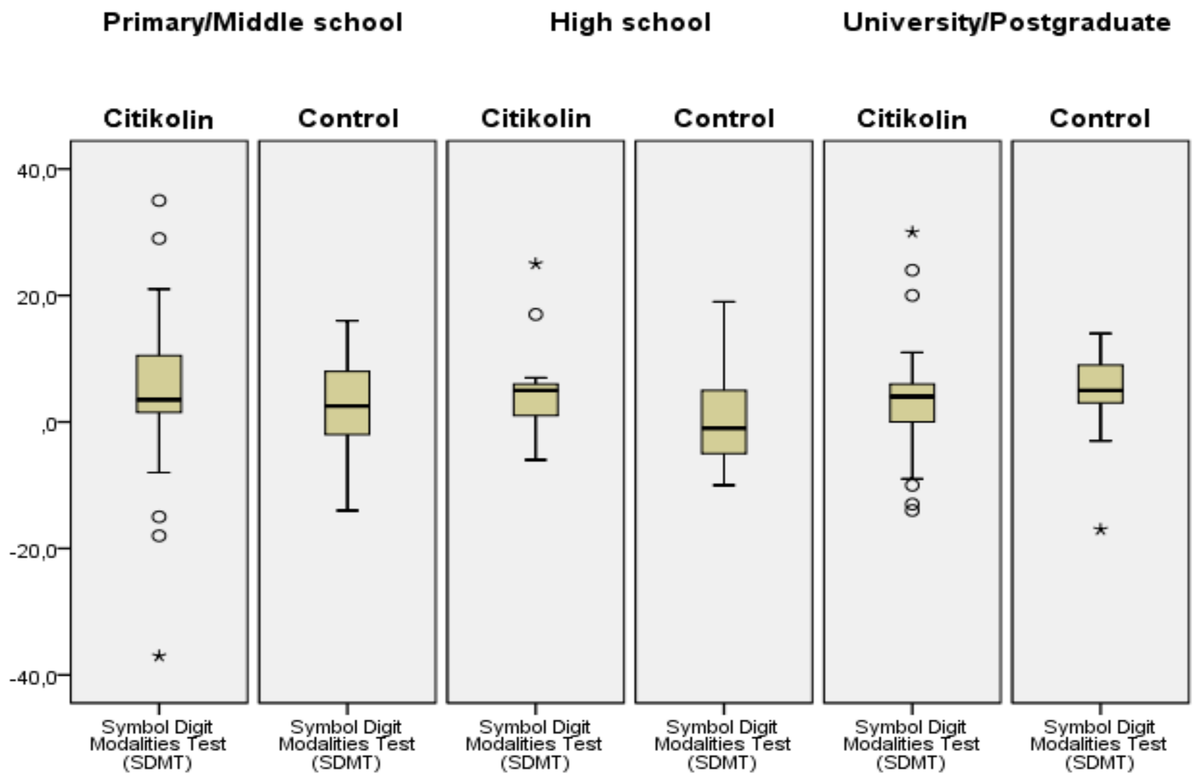

Figure S5. Changes in Semantic Fluency Scores According to Educational Level in Ci ticoline and Control Groups.

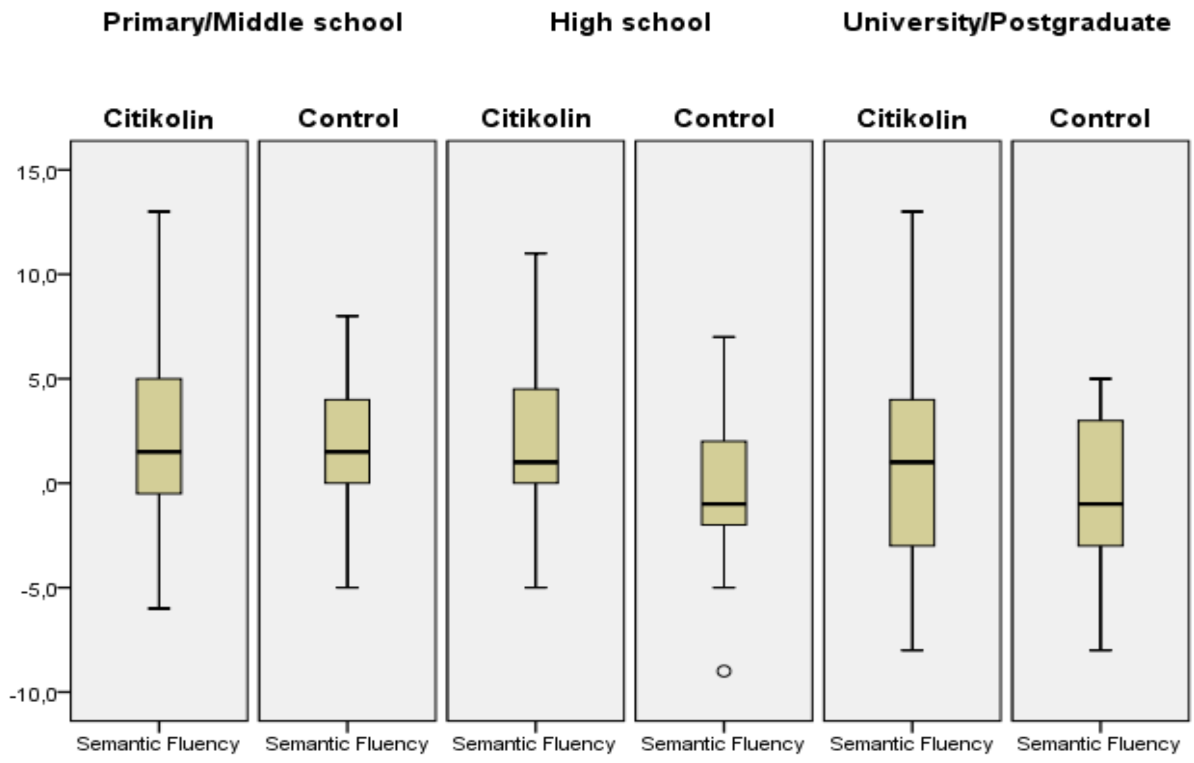

Figure S6. Changes in Short Blessed Test (SBST) Scores According to Educa tional Level in Citicoline and Control Groups.

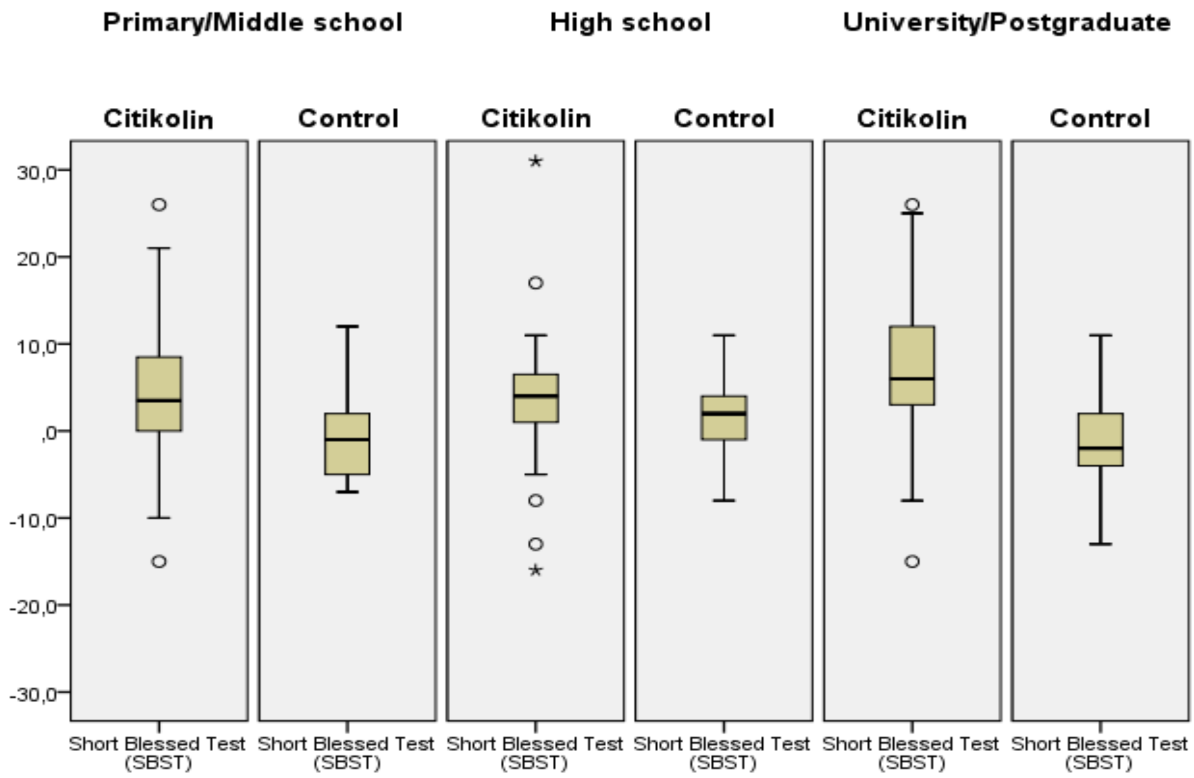

Figure S7. Changes in Short Blessed Test – Universal Stimuli Block Version (SBST-USB) Scores According to Educational Level in Citicoline and Control Groups.

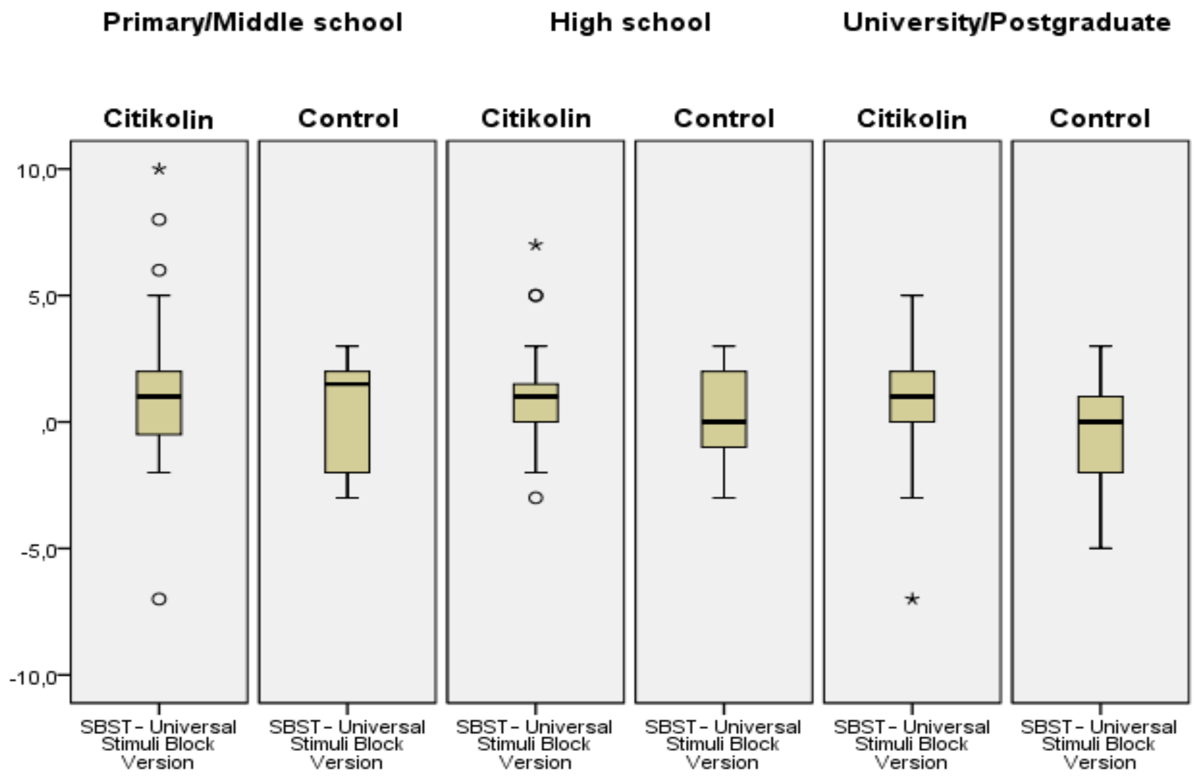

Figure S8. Changes in SPART – Universal Stimuli Block Version Scores According to Educational Level in Citicoline and Control Groups.

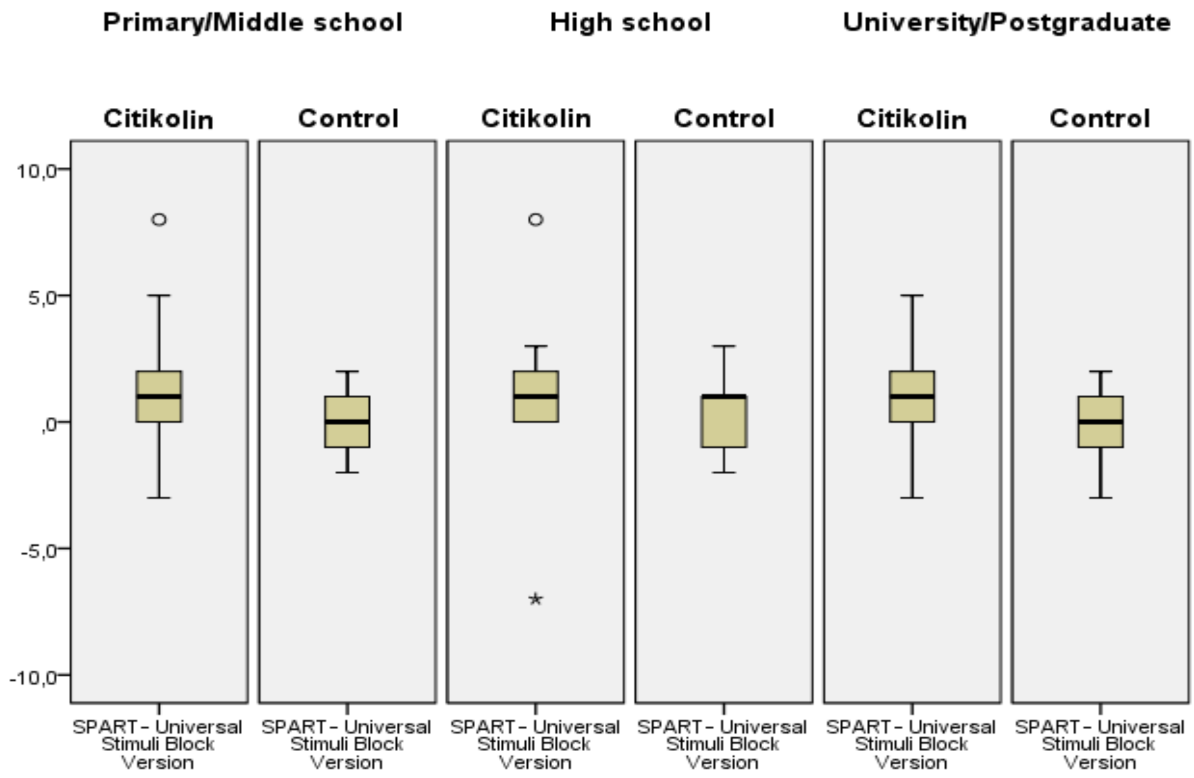

Figure S9. Changes in Episodic Fluency Scores According to Educational Level in Ci ticoline and Control Groups.

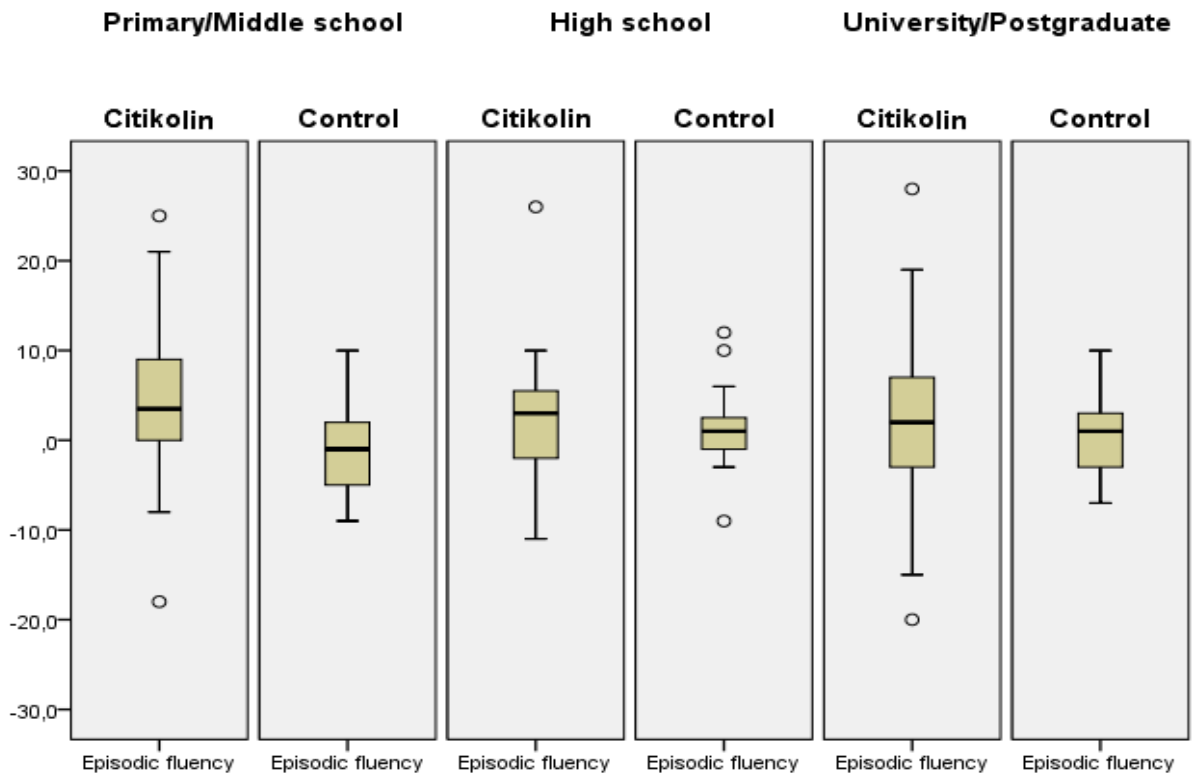

Figure S10. Changes in Boston Naming Test (BNT) Scores According to Educational Level in Citicoline and Control Groups.

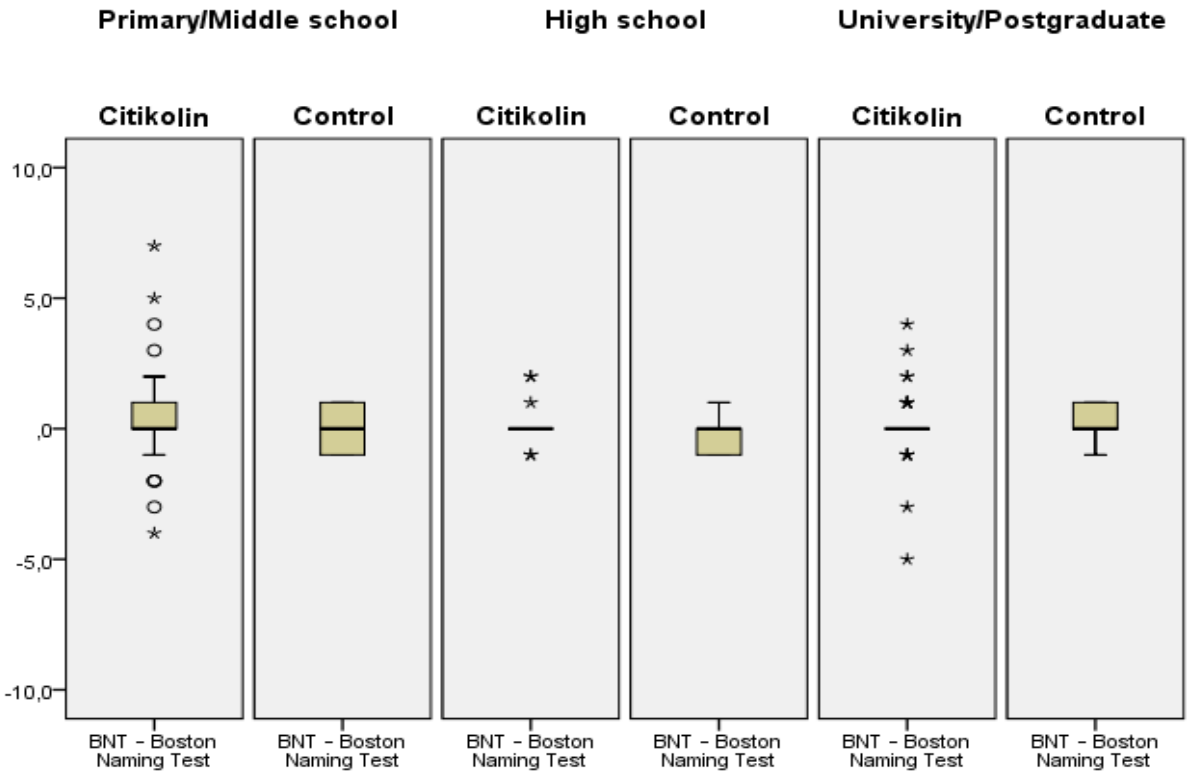

Figure S11. Changes in SPART Total Learning (SPART-TL) Scores According to Educa tional Level in Citicoline and Control Groups.

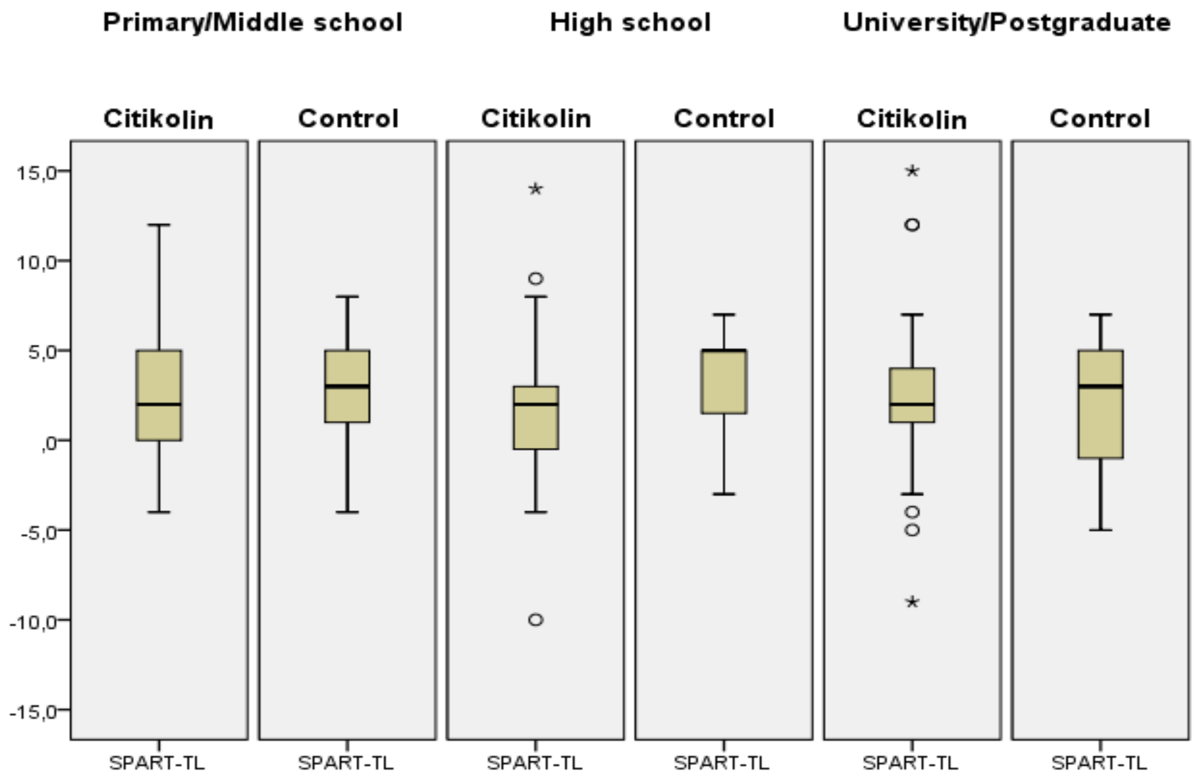

Table S1

1. Brief Repeatable Battery of Neuropsychological Tests (BRB-N)

A validated battery widely used across neurodegenerative disorders (Benedict 2002). Includes:

a. Spatial Recall Test (SPART) & SPART–Delayed Recall

- Measures visuospatial learning and short-term visual memory (Rao 1990).
- Immediate phase: three learning trials (0–36 points).
- Delayed recall: after 15 minutes, scored 0–12.
- Turkish validity: Bingöl et al. (2012).
- Sensitive to hippocampal/parietal dysfunction typical of early Alzheimer pathology.

b. Selective Reminding Test (SRT) & SRT–Delayed Recall

- Developed by Buschke (1973) and normed by Ehrenreich (1995)
- Evaluates verbal learning across **six recall trials (0–72 points)**.
- Delayed recall trial performed after **25 minutes (0–12 points)**.
- Measures long-term storage (LTS) and consistent long-term retrieval (CLTR).
- Turkish version: Bingöl et al. (2012).

c. Symbol Digit Modalities Test (SDMT)

- Measures processing speed, attention, and visual scanning (Smith 1968; 1973)
- Score range: number of correct symbols–digit pairings in **90 seconds**.
- One of the most sensitive markers of global cognitive slowing in early decline.
- Turkish norms: Aksoy et al. (2023).

d. Word List Generation (WLG)

- Semantic and phonemic fluency assessed using **animal names** and **K-A-S initial letters** (Bingöl et al., 2012).
- Based on Troyer et al.'s clustering/switching model (1997).
- Sensitive to frontotemporal network integrity.

2. Executive Functions and Attention Measures

Stroop Test – Çapa Version

- Examines selective attention and inhibition control (Golden 1978).
- Turkish adaptation: Emek-Savaş et al. (2020).
- Only Card 2 was used; time, errors, and spontaneous corrections were recorded.

Trail Making Test A and B (TMT-A/B)

- Classic measure of processing speed, cognitive flexibility, and set-shifting (Reitan 1955).
- Form A: numeric sequencing (processing speed).
- Form B: alternating numbers and letters (executive control).
- Turkish standardization: Cangöz et al. (2007).

3. Global Cognitive Screening – MoCA

- Assesses global cognition across executive, visuospatial, memory, language, abstraction, and orientation domains (Nasreddine 2005).
- Turkish version cutoff: 21/30 (Selekler et al. 2010)

4. Additional Cognitive Measures

Boston Naming Test (BNT)

- Measures confrontation naming and semantic retrieval (Kaplan 1983).
- Short form (BNT-30) used.
- Turkish norms: Ekinci (2016); Soylu & Cangöz (2018).

Clock Drawing Test (CDT)

- Assesses visuospatial construction, executive planning, and parietal–frontal integration (Shulman 2000).

WMS-III – Test of Mental Control

- Measures sequencing, attention, and rapid mental operations (Wechsler 1945; 1997).

- Turkish adaptation: Ant (2005).

5. Mood and Anxiety Measures

Beck Depression Inventory (BDI)

- 21-item measure of depressive symptoms (Beck 1961; Beck et al. 1996)
- Turkish adaptation: Hisli (1989).
- Cutoff of **10 points** used.

Beck Anxiety Inventory (BAI)

- 21-item scale measuring physical and cognitive anxiety (Beck 1988).
- Turkish adaptation: Ulusoy et al. (1998).
- Cutoff: **10 points**.

Geriatric Depression Scale (GDS-30)

- Developed by Yesavage et al. (1983) and adapted to Turkish by Ertan & Eker (2000)
- Cutoff: **≥5 points**.

Mood assessments were included because anxiety and depression significantly influence cognitive performance in prodromal dementia.

References

1. Benedict RHB, Fischer JS, Archibald CJ, et al. Minimal neuropsychological assessment of MS patients: A consensus approach. Clin Neuropsychol. 2002;16(3):381-397.
2. Bingöl, A., Yıldız, S., Topçular, B., Tutuncu, M., Demirci, N. O., Uygunoğlu, U., et al. (2012). Brief Repeatable Battery (BRB) – Turkish normative data. In Proceedings of the 16th Congress of the European Federation of Neurological Societies (EFNS), Stockholm, Sweden. European Journal of Neurology, 19, 55887–90.
3. Ehrenreich, J. H. (1995). Normative data for adults on a short form of the Selective Reminding Test. Psychological Reports, 76, 3.
4. Smith A. Symbol Digit Modalities Test manual. Los Angeles: Western Psychological Services; 1982. (WoS'ta "recorded item" olarak görünür—test manueli olduğu için sınırlı kayıt bulunur.)
5. Troyer AK, Moscovitch M, Winocur G. Clustering and switching as two components of verbal fluency: Evidence from younger and older adults. Neuropsychology. 1997;11(1):138–146.
6. Golden CJ. Stroop Color and Word Test: A manual for clinical and experimental uses. Chicago: Stoelting; 1978. (WoS'ta kayıtlıdır, atıf alan eser.)

7. Emek Savaş, D. D., Yerlikaya, D., Yener, G. G., & Öktem Tanör, Ö. (2020). Stroop Testi Çapa Formu'nun geçerlik-güvenirlik ve norm çalışması. Türk Psikiyatri Dergisi, 31(1), 9-21.
8. Reitan, R. M. (1955). The relation of the Trail Making Test to organic brain damage. Journal of Consulting Psychology, 19(5), 393–394. https://doi.org/10.1037/h0044509
9. Cangöz, B., Karakoç, E. ve Selekler, K. (2007). İz Sürme Testi'nin 50 yaş üzeri Türk yetişkin ve yaşlı örneklemi için standardizasyon çalışması. Türk Geriatri Dergisi, 10(2), 73-82. http://geriatri.dergisi.org/pdf/pdf_TJG_354.pdf
10. Nasreddine, Z. S., Phillips, N. A., Bédirian, V., Charbonneau, S., Whitehead, V., Collin, I., Cummings, J. L., & Chertkow, H. (2005). Montreal Cognitive Assessment (MoCA) [Database record]. APA PsycTests.
11. Selekler, K., Cangöz, B., & Uluç, S. (2010). Power of discrimination of the Montreal Cognitive Assessment (MoCA) scale in Turkish patients with mild cognitive impairment and Alzheimer's disease. Turkish Journal of Geriatrics, 13(1), 166–171.
12. Kaplan E, Goodglass H, Weintraub S. Boston Naming Test. Philadelphia: Lea & Febiger; 1983. (WoS'ta bibliyografik kayıt bulunmaktadır.)
13. Ekinci, A. (2016). Boston Adlandırma Testi'nin sağlıklı yaşlı örneklemde uyarlama ve norm belirleme çalışması[Yayınlanmamış yüksek lisans tezi]. Hacettepe Üniversitesi, Ankara.
14. Soylu, A. E., & Cangöz, B. (2018). Adaptation and Norm Determination Study of the Boston Naming Test for Healthy Turkish Elderly. Noro psikiyatri arsivi, 55(4), 341–348. https://doi.org/10.5152/npa.2017.19331
15. Shulman KI. Clock-drawing: Is it the ideal cognitive screening test? Int J Geriatr Psychiatry. 2000;15(6):548–561.
16. Wechsler D. Wechsler Memory Scale – Third Edition (WMS-III). San Antonio: Psychological Corporation; 1997.
17. Ant,S,E.,(2005).Wechsler Bellek Ölçeği-III' Sözel Çağrışım Çiftleri-I ve II, İşitsel Gecikmeli Tanıma alt testlerinin Türkçe geçerlilik, güvenirlik ön çalışması (Yayınlanmamış yüksek lisans tezi). Anadolu Üniversitesi Sağlık Bilimleri Enstitüsü, Eskişehir.

18. BECK, A. T., WARD, C. H., MENDELSON, M., MOCK, J., & ERBAUGH, J. (1961). An inventory for measuring depression. Archives of general psychiatry, 4, 561–571. https://doi.org/10.1001/archpsyc.1961.01710120031004
19. Ulusoy, M., Sahin, N. H., & Erkmen, H. (1998). Turkish version of the Beck Anxiety Inventory: Psychometric properties. Journal of Cognitive Psychotherapy, 12(2), 163–172.
20. Hisli, N. (1989). Beck Depresyon Envanterinin üniversite öğrencileri için geçerliği, güvenirliği. Psikoloji dergisi, 7(23), 3-13.
21. Beck, A. T., Epstein, N., Brown, G., & Steer, R. A. (1988). An inventory for measuring clinical anxiety: Psychometric properties. Journal of Consulting and Clinical Psychology, 56(6), 893–897. https://doi.org/10.1037/0022-006X.56.6.893
22. Ertan, T., & Eker, E. (2000). Reliability, validity, and factor structure of the geriatric depression scale in Turkish elderly: are there different factor structures for different cultures?. International psychogeriatrics, 12(2), 163–172. https://doi.org/10.1017/s1041610200006293

Table S2. Memory, language, global cognition, and emotional measures

Variable	**Citicoline (n=100) Median (Min–Max)**	**Citicoline Mean ± SD**	**Control (n=50) Median (Min–Max)**	**Control Mean ± SD**	**p-value**
Semantic Fluency – Baseline	16 (5–35)	15.72 ± 5.04	22 (18–32)	22.56 ± 3.09	<0.001
Semantic Fluency – Follow-up	17 (2–41)	17.29 ± 5.62	22 (19–28)	22.66 ± 2.45	<0.001
Episodic Fluency – Baseline	30 (10–63)	30.87 ± 10.73	44 (38–50)	43.78 ± 3.44	<0.001
Episodic Fluency – Follow-up	32.5 (8–59)	33.74 ± 10.88	43.5 (38–52)	44.00 ± 3.72	<0.001
SPART – Baseline	15 (6–29)	15.63 ± 5.28	22 (18–29)	22.34 ± 2.86	<0.001
SPART – Follow-up	18.5 (5–30)	18.25 ± 5.47	23 (19–29)	22.94 ± 2.53	<0.001

SPART-USB – Baseline	5 (0–10)	4.66 ± 2.30	8 (6–10)	7.72 ± 1.05	<0.001
SPART-USB – Follow-up	6 (0–10)	5.63 ± 2.23	8 (6–10)	7.98 ± 0.96	<0.001
SBST – Baseline	42.5 (14–131)	49.76 ± 27.08	55 (46–65)	55.54 ± 4.27	<0.001
SBST – Follow-up	48 (15–142)	55.31 ± 29.20	55 (48–65)	55.32 ± 3.53	<0.001
Boston Naming Test – Baseline	29 (15–30)	27.68 ± 3.21	30 (29–30)	29.74 ± 0.44	<0.001
Boston Naming Test – Follow-up	30 (12–30)	28.00 ± 3.07	30 (29–30)	29.74 ± 0.44	<0.001
MoCA – Baseline	22 (8–29)	21.99 ± 4.29	28 (24–30)	27.72 ± 1.28	<0.001
MoCA – Follow-up	24 (8–30)	23.34 ± 4.30	28 (25–30)	27.74 ± 1.14	<0.001
Beck Depression – Follow-up	5 (4–16)	6.88 ± 3.76	10 (4–16)	10.52 ± 2.67	<0.001
Beck Anxiety – Follow-up	7 (0–22)	7.74 ± 4.99	5.5 (0–11)	5.52 ± 3.44	0.018

Higher scores indicate better performance for cognitive tests, except for mood scales, where lower scores reflect improvement.
